# Statin Use and the Risk of Cataracts: A Systematic Review and Meta‐Analysis

**DOI:** 10.1161/JAHA.116.004180

**Published:** 2017-03-20

**Authors:** Shandong Yu, Yanpeng Chu, Gang Li, Lu Ren, Qing Zhang, Lin Wu

**Affiliations:** ^1^ Department of Cardiology Peking University First Hospital Beijing China

**Keywords:** cataract, meta‐analysis, observational studies, randomized controlled trial, statin, Meta Analysis

## Abstract

**Background:**

Cataracts are the main cause of poor vision and blindness worldwide. The effects of statin administration on cataracts remain debated. Therefore, we conducted a systematic review and meta‐analysis to determine whether statin use affects the risk of cataracts.

**Methods and Results:**

We performed a systematic search of the electronic databases PubMed, EMBASE, and the Cochrane Library through January 2016. Weighted averages were reported as relative risk values with 95% CIs. Statistical heterogeneity scores were assessed with the standard Cochran's Q test and the I^2^ statistic. A total of 6 cohort studies, 6 case–control studies, and 5 randomized controlled trials, together involving more than 313 200 patients, were included in our study. The pooled estimates of cohort studies indicated that the use of statins moderately increases the risk of cataracts (relative risk, 1.13; 95% CI, 1.01–1.25). The pooled estimates of case–control studies (relative risk=1.10, 95% CI, 0.99–1.23) and randomized controlled trials (relative risk, 0.89; 95% CI, 0.72–1.10) indicated that the use of statins does not increase the risk of cataracts. The sensitivity analysis confirmed the stability of the results. Heterogeneity was found among the cohort and case–control studies.

**Conclusions:**

Based on the present meta‐analysis of these studies, we could only conclude that there is no clear evidence showing that statin use increases the risk of cataracts. The most likely case is that there is no association between statin use and cataracts. Because of the considerable benefits of statins in cardiovascular patients, this issue should not deter their use.

## Introduction

Cataracts are the main cause of low vision and blindness worldwide.^1^ Nearly 13 million people in the United States are reported to suffer from cataracts.^2^ Statins are widely prescribed to treat hyperlipidemia, as they reduce the risk of cardiovascular disease by inhibiting 3‐hydroxy‐3‐methyl‐glutaryl‐CoA reductase.^3^, ^4^ Although it has been established that statins can significantly reduce cardiovascular mortality,^5^, ^6^, ^7^, ^8^ some adverse effects related to statins have been recognized because of their increasing use.^9^, ^10^

Concern about the cataractogenic effect of statins arose from animal studies in which dogs were administered high doses of statins, such as simvastatin, fluvastatin, and lovastatin.^10^, ^11^ However, in human studies, investigations into the association between statin use and the incidence of cataracts and cataract surgery have yielded inconsistent and conflicting results.^12^, ^13^, ^14^, ^15^, ^16^, ^17^, ^18^, ^19^, ^20^, ^21^, ^22^ Some studies have reported no association between statin use and cataracts,^12^, ^13^, ^14^, ^23^, ^24^, ^25^, ^26^ whereas others have found that statin use is protective against the incidence of cataracts,^15^, ^16^, ^27^ or that it is associated with an increased risk of cataracts.^17^, ^18^, ^19^, ^20^, ^21^, ^22^ To address this issue, Kostis et al^28^ performed a meta‐analysis in 2013. However, they combined the unadjusted odds ratio directly without considering potential confounding factors in some studies, which may have led to inaccurate results. In addition, some studies have been published in the 2 years since the study by Kostis et al^28^ was published, of which most yielded results that conflicted with those included in Kostis et al.^18^, ^19^, ^21^, ^22^ Therefore, we performed a new meta‐analysis to investigate the association between statin use and cataracts.

## Methods

We conducted this meta‐analysis following the guidance provided by the Cochrane Handbook^29^ and Kanwal and White.^30^ Two authors (Yu and Chu) independently performed the literature search, article screening, study selection, quality evaluation, and data extraction. Disagreements were resolved by discussion, and a consensus was achieved in the selection of the articles for analysis.

### Search Strategy

The Cochrane Library, PubMed, and EMBASE databases were searched from January 1980 to January 2016 for English language publications, including abstracts. The search was performed using the following terms: “statins OR HMG‐CoA reductase inhibitors OR Simvastatin OR Lovastatin OR Fluvastatin OR Pravastatin OR Rosuvastatin OR Atorvastatin” AND “cataract.” We also manually searched for relevant articles from the reference lists of the retrieved articles. When the available information was incomplete, we attempted to contact the study investigators for additional information.

### Study Selection

Studies were included in this meta‐analysis if they fulfilled the following criteria: (1) the study was a case–control, cohort study, or randomized controlled trials (RCTs); (2) non–statin users were included in the comparison group; (3) cataracts and/or cataract surgery was an outcome; (4) the association between statin use and the risk of cataracts/cataract surgery was investigated; (5) risk estimates of morbidity and 95% CIs were reported or the information required to calculate them was available. Basic science studies, reviews, editorials/letters, case reports, and studies without comparison groups were excluded.

### Data Extraction and Quality Assessment

Data extraction was performed independently by 2 of the authors (Yu and Chu). The following information was extracted from each study: the last name of the first author, year of publication, study design, country of origin of the population studied, patient characteristics, statin use, information source for exposure ascertainment, risk estimates and corresponding 95% CIs, and covariates adjusted for in the multivariable analysis. For studies that provided more than 1 risk estimate, we extracted the estimate that was adjusted for the greatest number of confounding factors. We assessed the methodological quality of the included studies based on the Newcastle‐Ottawa Scale (NOS) for observational studies,^31^ which was developed to assess the quality of nonrandomized studies in meta‐analysis. Using this scale, observational studies were scored across 3 categories as follows: selection (4 questions) and comparability (2 questions) of the study group and ascertainment of the outcome of interest (3 questions), with all questions having a score of 1 except for the comparability of study groups, for which separate points were awarded for controlling for age and/or sex (maximum, 2 points). A score of ≥7 points was suggestive of a high‐quality study. The quality of the included RCTs was assessed by Cochrane risk of bias assessment,^29^ which allots scores for the following: random sequence generation (1), allocation concealment (1), blinding of participants and personnel (1), blinding of outcome assessment (1), incomplete outcome data (1), selective reporting (1), and other sources of bias (1). Scores of 1 to 4 indicate low quality, and scores of 5 to 7 indicate high quality.

### Outcomes Assessed

The primary analysis focused on assessing the risk of cataracts and cataract surgery among users of statins. We also performed subgroup analyses based on study design (case–control, cohort, or RCT), type of statin, the methodological quality of the study (high or low), study location (Europe, North America, Asia or Australia), age, sex, follow‐up duration, outcome and outcome assessment, and whether potential confounders were included in the adjusting model (eg, low‐density lipoprotein included/missing, cardiovascular disease [CVD] included/missing, smoking included/missing).

### Statistical Analysis

STATA 12.0 software (StataCorp LP, College Station, TX) was used for statistical analysis. Heterogeneity was assessed using the Cochran Q χ^2^ test and the I^2^ statistic.^32^ An I^2^ value of >50% or a *P* value of <0.05 for the Q‐statistic indicated significant heterogeneity.^33^ Adjusted effect estimates (odds ratios, relative risks [RRs], and hazard risks) between the outcome and use of statins were extracted. In the presence of heterogeneity, we used a random‐effects model because its assumptions account for the presence of variability among studies. The association between statin use and cataract or cataract surgery risk was estimated using the RRs and corresponding 95% CIs. Because the outcomes were relatively uncommon and the odds ratios in the case–control studies were close to 1, odds ratios were considered approximations of RR.^34^

## Results

### Search Results

**Figure 1:**
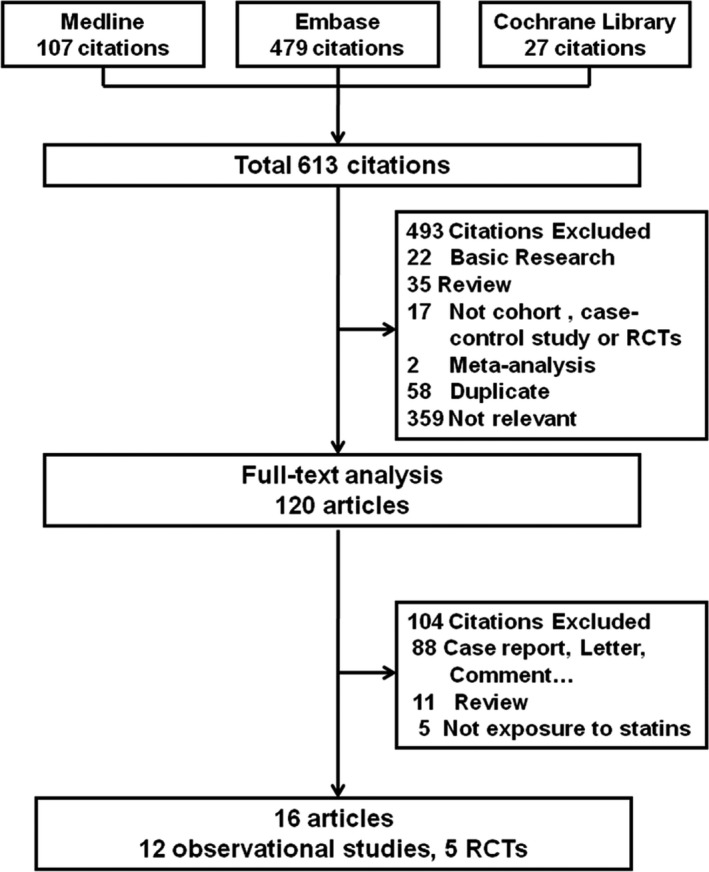
Flow chart of the studies considered and selected for review. RCTs indicates randomized controlled trials.

By searching the 3 databases, 615 potentially eligible articles were identified. In total, 489 articles were excluded after reading the title and abstract, and the full texts of the remaining 120 articles were evaluated in detail. Of these 120 articles, 16 met our inclusion criteria. One of these articles included 2 studies.^21^ In total, 17 studies consisting of 6 cohort studies, 6 case–control studies, and 5 RCTs were included in the meta‐analysis and involved more than 313 200 cataract cases. The number of articles according to reason for exclusion at each stage of the eligibility assessment is outlined in Figure 1.

### Characteristics of the Included Studies

The main characteristics of the cohort and case–control studies are shown in Tables 1 and 2. Among the cohort studies, 3 studies were performed in North America, and the remaining 3 studies were performed in Europe, Asia, and Australia.^15^, ^16^, ^17^, ^18^, ^19^, ^20^ Among the case–control studies, 4 studies were performed in North America, and 2 were performed in Europe.^12^, ^13^, ^14^, ^21^, ^22^ The extent of adjustment for potential clinical risk factors varied considerably across the cohort and case–control studies (Table S1). Based on the methodological quality assessment scores (Tables S2 and S3), the mean score of the 6 cohort studies included in the analysis was 7. Four studies were of high quality (NOS ≥7), and 3 studies were of low quality (NOS <7). The mean score of the 6 case–control studies was 6.5. Three studies were of high quality (NOS ≥7), and 3 studies were of low quality (NOS <7). The characteristics of the RCTs are shown in Table 3.^23^, ^24^, ^25^, ^26^, ^27^ Two trials were performed in the United States, and 3 trials were performed in Europe. The mean score of the RCTs included in the analysis was 5.4 (Table S4).

**Table 1:** Characteristics of the Cohort Studies

Study, Year	Location	Follow‐Up Period, y	Age (Mean), y	Outcome	Outcome Assessment	Ascertainment of Statins Exposure	Cases, n	Controls, n	Overall Quality
Klein, 2006^15^	US	5	63.2	Cataract	Wisconsin Cataract Grading System	Private census	185/834	42/214	7
Tan, 2007^16^	AUS	>5	≥50 (>60)	Cataract Cataract surgery	Wisconsin Cataract Grading System	Questionnaires	17/72	365/1152	6
Hippisley‐Cox, 2010^17^	UK	<5	30 to 84 (<60)	Cataract	Medical records	Drug prescription Computerized record	36 541	2 004 692	8
Waudby, 2011^20^	US	>5	>60	Cataract Cataract surgery	ICD‐9 CPT code	Drug prescription Computerized record	1959/7470	1645/12 579	6
Lai, 2013^19^	Asia	>5	70.4	Cataract surgery	ICD‐9	Drug prescription Computerized record	1533/6830	16 137/43 335	8
Leuschen, 2013^18^	US	<5	30 to 85 (<60)	Cataract	ICD‐9	Drug prescription Computerized record	2477/6972	2337/6972	7

CPT indicates current procedural terminology; ICD, International Classification of Diseases.

**Table 2:** Characteristics of the Case–Control Studies

Study, Year	Location	Follow‐Up Period, y	Age (Mean), y	Outcome	Outcome Assessment	Ascertainment of Statins Exposure	Cases, n	Controls, n	Overall Quality
Schlienger, 2001^12^	UK	<5	40 to 79 (>60)	Cataract Cataract surgery	ICD‐8, OMXIS procedure code 156	Drug prescription Computerized record	7405	28 327	7
Smeeth, 2003^13^	UK	<5	≥40 (75)	Cataract	ICD‐8	Drug prescription Computerized record	15 479	15 479	7
Fong, 2012^14^	US	<5	≥50 (>60)	Cataract surgery	CPT code	Drug prescription Computerized record	13 583	34 049	6
Wise‐BC, 2014^21^	Canada	<5	>70	Cataract Cataract surgery	Computerized record	Drug prescription Computerized record	162 501	650 004	6
Wise‐IMS, 2014^21^	Canada	<5	40 to 85 (>70)	Cataract Cataract surgery	Computerized record	Drug prescription Computerized record	45 065	450 650	7
Erie, 2016^22^	US	<5	≥50 (>60)	Cataract Surgery	ICD‐9 CPT code	Drug prescription Computerized record	6024	6024	6

CPT indicates current procedural terminology; ICD, International Classification of Diseases; OMXIS, Oxford Medical Information System.

**Table 3:** Characteristics of RCTs

Study, Year	Location	Study Design	Follow‐Up Period	Age (Mean), y	Outcome	Outcome Assessment	Type of Statins	Cases/Statins Group	Cases/Control Group
Laties, 1991^23^	US	RCT	48 weeks	18 to 70 (<60)	Cataract surgery	Slit‐lamp examination	Lovastatin	25/6582	7/1663
Harris, 1995^24^	EU	RCT	18 months	40 to 75 (>60)	Cataract and Cataract surgery	OXGRAE	Simvastatin	30/414	10/207
Pederson, 1996^25^	EU	RCT	5.4 years	35 to 70 (>60)	Cataract	Slit‐lamp examination	Simvastatin	66/2221	53/2223
Heart protection study, 2002^26^	US	RCT	5 years	40 to 80 (NA)^a^	Cataract	Medical records	Simvastatin	393/10 232	404/10 237
Bang, 2015^27^	US	RCT	4.3 years	45 to 85 (>60)	Cataract	Medical records	Simvastatin plus Ezetimibe	NA/944^b^	NA/929^b^

RCTs indicates randomized controlled trials; NA indicates not available; OXGRADE, Oxford grading system.

a The authors did not show the mean age of subjects.

b The authors showed the relative risk value instead of number of cases.

### Main Analysis

**Figure 2:**
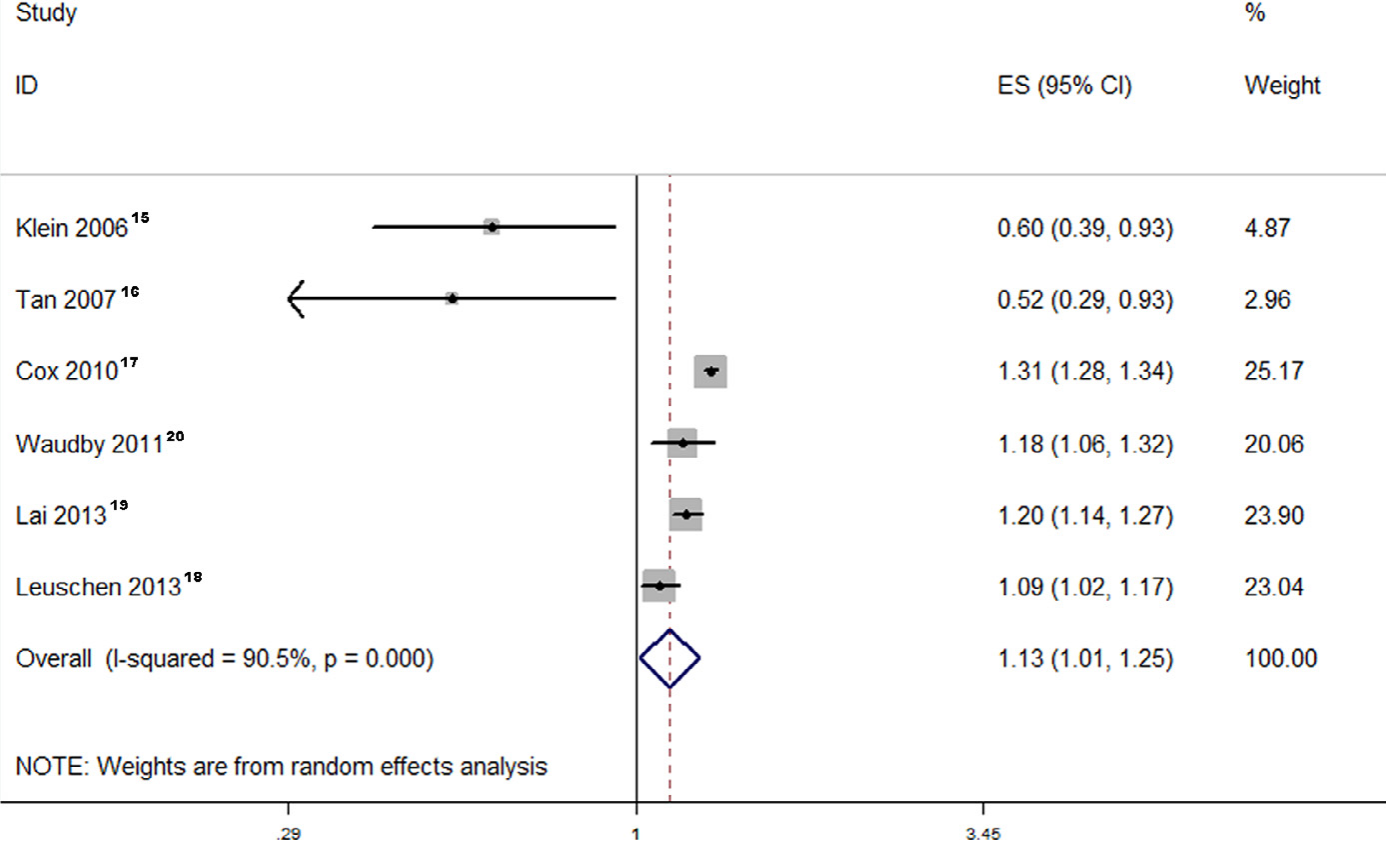
Forest plot of the cohort studies. The pooled RR of the cohort studies was 1.13 (95% CI, 1.01–1.25). The I^2^ value indicated considerable heterogeneity across these cohort studies (I^2^=90.5%; *P*<0.001). ES indicates effect size; RR, relative risk.

**Figure 3:**
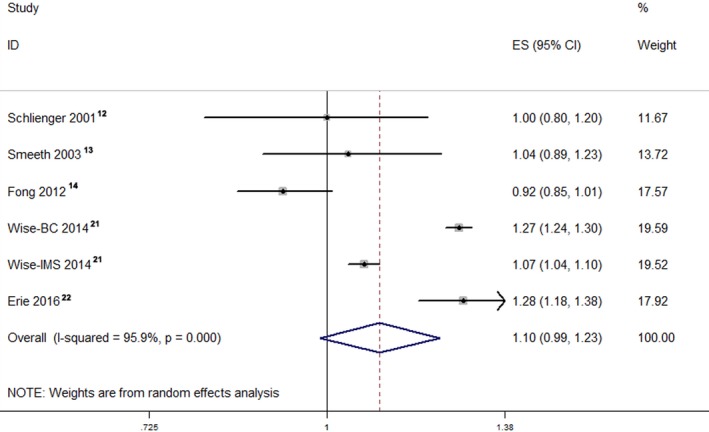
Forest plot of the case–control studies. The pooled RR of the case–control studies was 1.10 (95% CI, 0.99–1.23). The I^2^ value revealed considerable heterogeneity across these case–control studies (I^2^=95.9%; *P*<0.001). ES indicates effect size; RR, relative risk.

**Figure 4:**
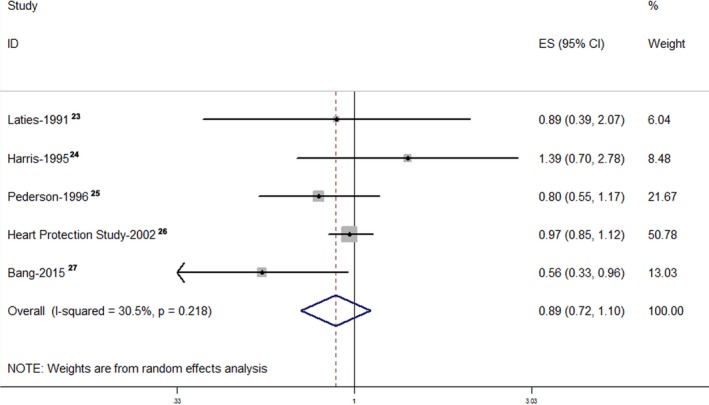
Forest plot of the RCTs. The pooled RR of the RCTs was 0.89 (95% CI, 0.72–1.10). The I^2^ value indicated slight heterogeneity across these RCTs (I^2^=30.5%; *P*=0.218). ES indicates effect size; RCT, randomized controlled trial; RR, relative risk.

The pooled RR of the cohort studies was 1.13 (95% CI, 1.01–1.25), which indicated that the use of statins was associated with a 13% increase in cataract incidence or cataract surgery (Figure 2). The I^2^ value indicated significant heterogeneity across the studies (I^2^=90.5%; *P*<0.001). However, the pooled RRs of the case–control studies and RCTs were 1.10 (95% CI, 0.99–1.23) (Figure 3) and 0.89 (95% CI, 0.72–1.10) (Figure 4),^23^, ^24^, ^25^, ^26^, ^27^ which indicated that the use of statins was not associated with cataract incidence or cataract surgery. The I^2^ value of the case–control studies was 95.9%, which indicated significant heterogeneity across the studies. However, the I^2^ value of the RCTs was 30.5%, which indicated low heterogeneity across the RCTs. We also performed a sensitivity analysis for the cohort studies (Figure S1), case–control studies (Figure S2), and RCTs (Figure S3), and the results showed that the sequential omission of individual studies did not alter the overall effect. The pooled estimate effect size changed from 1.07 (95% CI, 0.91–1.25) to 1.17 (95% CI, 1.06–1.25) for the cohort studies, from 1.06 (95% CI, 0.96–1.19) to 1.14 (95% CI, 1.03–1.28) for the case–control studies, and from 0.82 (95% CI, 0.58–1.15) to 0.96 (95% CI, 0.85–1.09) for the RCTs.

### Subgroup Meta‐Analyses

The results of the subgroup analyses of the cohort and case–control studies are presented in Tables S5 and S6. In the subgroup analysis of cohort studies, there were significant associations in the subgroups of high methodological quality, outcome assessment, cataract, no older than 60, less than 5 years follow‐up duration, low‐density lipoprotein missing model, CVD included model, consultation rate included model, and hypertension included model. No associations were observed in the low methodological quality, studies performed in North America, older than 60 years, more than 5 years follow‐up duration, low‐density lipoprotein included model, CVD missing model, smoking missing model, consultation rate missing model, and hypertension missing model subgroups (Table S5, Figures S4 through S15). In the subgroup analysis of case–control studies, significant associations were observed in the subgroups of atorvastatin, lovastatin, high methodological quality, cataract surgery, CVD included model, smoking missing model, consultation rate missing model, and hypertension included model. No associations were observed in the fluvastatin, rosuvastatin, pravastatin, simvastatin, low methodological quality, studies performed in North America and Europe, outcome assessment, cataract and cataract surgery, CVD missing model, smoking included model, consultation rate included model, and hypertension missing model subgroups (Table S6, Figures S16 through S25).

The results of the subgroup analysis of RCTs are presented in Table S7.

When the studies were grouped according to patient age, no association was observed in the older than 60 years subgroup (RR, 0.82; 95% CI, 0.53–1.26) and the no older than 60 years subgroup (RR, 0.97; 95% CI, 0.84–1.11) (Figure S26). Although the mean age of the Heart Protection Study subjects was not presented, the results did not change regardless of which group we placed this study in (Figure S27).

When we grouped the studies by follow‐up duration, no significant association was observed in the no more than 5 years group (RR, 0.86; 95% CI, 0.48–1.51) and in the more than 5 years group (RR, 0.95; 95% CI, 0.83–1.08) (Figure S28).

## Discussion

In this comprehensive meta‐analysis of 6 cohort, 6 case–control studies, and 5 RCTs, we analyzed the effect of statin use on the risk of cataracts in more than 313 200 patients. Analysis of the cohort studies showed that statin use was associated with a 13% increased risk of cataracts. However, analysis of the case–control studies and RCTs revealed no association between statin use and the risk of cataract. The effect size of the case–control studies was marginal, namely, RR=1.10 (95% CI, 0.99–1.23). Based on the differing characteristics of observational (case–control and cohort) studies and RCTs, such discordant results are not unexpected. Because of the rigorous criteria of RCTs, individuals at greatest risk for adverse events may be excluded. Furthermore, the subjects of RCTs may be healthier than the subjects of observational studies.^35^ The RCTs in this analysis had good internal validity, but the external validity was limited. The conclusion could not be extended to the whole population. In a population similar to the study population, the conclusion was reliable. Moreover, there may be a large portion of patients similar to the patients enrolled in these RCTs. However, there are also many patients who are not similar to the patients enrolled in these RCTs. The observational studies involve more cases with different health conditions. However, in observational studies, baseline confounders can be present, which may affect the results. In such studies, relative to non–statin users, statin users may be expected to be of poorer health or to have higher risk factors that necessitate statin therapy. As a result, adverse event rates may be higher among statin users. Although most observational studies (including the present meta‐analysis) have attempted to characterize their patients and identify validated markers of morbidity and mortality, potential unidentified confounders may exist.^35^, ^36^ This may lead to a calculated effect size that is slightly higher than the real one. Therefore, the real effect may be no significant association.

The analyses of cohort and case–control studies were limited by the considerable heterogeneity across studies. In the subgroup analysis of cohort studies, the I^2^ values decreased significantly when subgrouped by sex, outcome assessment, age, follow‐up duration, or consultation rate included/missing model (Table S5, Figures S6, S7, S9, S10, and S14). In the female (Figure S6B), no older than 60 years (Figure S9B), and less than 5 years follow‐up subgroups (Figure S10B), the I^2^ values decreased because the weight of Cox's study was much higher (more than 70%). In the consultation rate included model subgroup (Figure S14A), the I^2^ value decreased because the weight of Lai's study was much higher (more than 80%). Consequently, the heterogeneity may be partly attributed to the outcome assessment. The evaluation criterion of various assessment methods may have varied among the studies, and patients diagnosed with cataracts by 1 method may not be so diagnosed when another method is used. Furthermore, even when the same method for diagnosis is used, different physicians may make different decisions, especially regarding cataract surgery. In the subgroup analysis of case–control studies, the I^2^ values were significantly decreased when subgrouped by quality assessment, study location, type of statin, CVD included/missing model, smoking included/missing model, consultation rate included/missing model, or hypertension included/missing model (Table S6, Figures S16, S17, S19, S21, S22, S23, and S25). In the quality assessment and hypertension included/missing model subgroups, the I^2^ values of the high quality group (Figure S16A) and the hypertension missing model (Figure S25B) decreased because the weights of the Wise‐IMS study (more than 95%) and the Fong study (more than 70%) were much higher than those of the other studies. In the subgroup analyses of the study performed in Europe (Figure S17B), the CVD missing model (Figure S21B), and the consultation rate included model (Figure S23A), the I^2^ values decreased because the included studies were derived from the same database. Therefore, the heterogeneity may be partly attributed to the types of statins. Statins have extensive pleotropic effects that extend beyond their cholesterol‐lowering properties.^35^, ^37^ Different types of statins may affect cataract development by different mechanisms. Therefore, patients taking different statins may have different risks for developing cataracts. In our subgroup analysis based on statin type, the I^2^ values of fluvastatin and pravastatin were significantly decreased compared with that of the overall result (Figure S19). Furthermore, the dose of statins also differed among studies. In addition to the fact that these factors may contribute to the heterogeneity, some other factors, such as ethnicity,^14^ ultraviolet exposure, and education level, may also lead to heterogeneity.^38^, ^39^, ^40^ The difference in the ascertainment method of statin use was also a source of heterogeneity. Klein et al^15^ and Tan et al^16^ determined statin use according to patient interviews, whereas in other studies, statin use was ascertained according to computerized prescription records.^12^, ^13^, ^14^, ^17^, ^18^, ^19^, ^20^, ^21^, ^22^ However, even if prescription records or interviews showed that a patient was prescribed statins, differences in patient compliance may have resulted in different degrees of exposure, which may have led to heterogeneity. Some previous studies have found that statin use has different effects on different types of cataract;^35^, ^37^ therefore, heterogeneity may result from study variations in the types of cataract and the proportions of statin types used.

Two of the included studies reported that statin use was protective against cataracts.^15^, ^16^ These 2 studies are long‐term prospective cohort studies that followed patients using periodical lens photographs. Such a design tends to achieve reliable results. However, these studies had limitations. The rate of loss to follow‐up was relatively high in these 2 studies (more than 20% at the 5th year).^15^, ^16^ Moreover, the sample sizes of these 2 studies were relatively small.

The analysis of the RCTs indicated that statin use does not increase the risk of cataract. Most of the individual results of included studies are consistent with this overall result. In the subgroup analyses by age and follow‐up duration, no association was observed between statin use and cataract risk (Table S7). The SEAS study reported that patients with aortic stenosis that were treated with simvastatin and ezetimibe had a lower risk of cataract than did patients treated with placebo.^27^ Because the treatment group received ezetimibe, which is a cholesterol‐lowering agent, this result may be overlooked in this study.^41^ Heterogeneity may have also arisen from this study.

The strengths of our meta‐analysis include the analysis of both observational studies and RCTs and the large sample size. Despite its strengths, there are several limitations of our analysis. First, evidence of among‐study heterogeneity of the observational studies was apparent. Although we performed subgroup analyses in an attempt to identify the sources of heterogeneity, these variables could not fully explain the observed heterogeneity, suggesting that other unknown, confounding variables might be responsible. Second, the confounding factors varied among the included studies.

Because of the limitations of observational studies and RCTs, large, multicenter, pragmatic, prospective observational studies or registries should be performed in the future to assess the risk of cataracts. The primary end points should include not only cardiovascular diseases but also total comorbidity. Moreover, patients should be stratified according to baseline confounders. Cataracts should be confirmed by objective serial testing using validated tools, and per‐protocol analysis should be used to determine the protocol effects on results. Finally, investigators should attempt to characterize and follow the outcomes of those patients who drop out of the trials.^35^

## Conclusion

Based on the present meta‐analysis of these studies, we could only conclude that there is no clear evidence showing that statin use increases the risk of cataract. The most likely case is that there is no association. Because of the considerable benefits of statins in cardiovascular patients, this issue should not deter the use of statins.

## Disclosures

None.

## Supporting information


**Table S1.** Detailed Definition of Cataract and Adjustment Factors for Cataract of Observational Studies
**Table S2.** NOS for Assessment of Quality of Included Studies: Case–Control Studies
**Table S3.** NOS for Assessment of Quality of Included Studies: Cohort Studies
**Table S4.** Quality of the Included RCTs Assessed by Cochrane Risk of Bias Assessment
**Table S5**. Subgroup analysis of cohort studies
**Table S6**. Subgroup analysis of case‐control studies
**Table S7**.Subgroup analysis of RCTs
**Figure S1.** Sensitivity analysis of cohort studies.
**Figure S2.** Sensitivity analysis of case–control studies.
**Figure S3.** Sensitivity analysis of RCTs.
**Figure S4.** Subgroup analysis by quality assessment .
**Figure S5.** Subgroup analysis by location (North America).
**Figure S6.** Subgroup analysis by sex.
**Figure S7.** Subgroup analysis by outcome assessment.
**Figure S8.** Subgroup analysis by outcome (Cataract).
**Figure S9.** Subgroup analysis by age.
**Figure S10.** Subgroup analysis by follow‐up duration.
**Figure S11.** Subgroup analysis by whether LDL included.
**Figure S12.** Subgroup analysis by CVD.
**Figure S13.** Subgroup analysis by smoking (smoking included).
**Figure S14.** Subgroup analysis by consultation rate.
**Figure S15.** Subgroup analysis by hypertension.
**Figure S16.** Subgroup analysis by quality assessment.
**Figure S17.** Subgroup analysis by location.
**Figure S18.** Subgroup analysis by outcome assessment.
**Figure S19.** Subgroup analysis by type of statins.
**Figure S20.** Subgroup analysis by outcome.
**Figure S21.** Subgroup analysis by CVD.
**Figure S22.** Subgroup analysis by smoking.
**Figure S23.** Subgroup analysis by consultation rate.
**Figure S24.** Subgroup analysis by diabetes mellitus (diabetes mellitus included).
**Figure S25.** Subgroup analysis by hypertension.
**Figure S26.** Subgroup analysis by age (older than 60).
**Figure S27.** Subgroup analysis by age (including Spence's).
**Figure S28.** Subgroup analysis by follow‐up duration.
