# Supplementary material for: Statin Use and the Risk of Cataracts: A Systematic Review and Meta‐Analysis
Source: J Am Heart Assoc. 2017 Mar 20;6(3):e004180. doi: 10.1161/JAHA.116.004180 (PMC5523994; doi:10.1161/JAHA.116.004180)
Supplement: Supplementary file 1 — Table S1. Detailed Definition of Cataract and Adjustment Factors for Cataract of Observational Studies Table S2. NOS for Assessment of Quality of Included Studies: Case–Control Studies Table S3. NOS for Assessment of Quality of Included Studies: Cohort Studies Table S4. Quality of the Included RCTs Assessed by Cochrane Risk of Bias Assessment Table S5. Subgroup analysis of cohort studies Table S6. Subgroup analysis of case‐control studies Table S7.Subgroup analysis of RCTs Figure S1. Sensitivity analysis of cohort studies. Figure S2. Sensitivity analysis of case–control studies. Figure S3. Sensitivity analysis of RCTs. Figure S4. Subgroup analysis by quality assessment . Figure S5. Subgroup analysis by location (North America). Figure S6. Subgroup analysis by sex. Figure S7. Subgroup analysis by outcome assessment. Figure S8. Subgroup analysis by outcome (Cataract). Figure S9. Subgroup analysis by age. Figure S10. Subgroup analysis by follow‐up duration. Figure S11. Subgroup analysis by whether LDL included. Figure S12. Subgroup analysis by CVD. Figure S13. Subgroup analysis by smoking (smoking included). Figure S14. Subgroup analysis by consultation rate. Figure S15. Subgroup analysis by hypertension. Figure S16. Subgroup analysis by quality assessment. Figure S17. Subgroup analysis by location. Figure S18. Subgroup analysis by outcome assessment. Figure S19. Subgroup analysis by type of statins. Figure S20. Subgroup analysis by outcome. Figure S21. Subgroup analysis by CVD. Figure S22. Subgroup analysis by smoking. Figure S23. Subgroup analysis by consultation rate. Figure S24. Subgroup analysis by diabetes mellitus (diabetes mellitus included). Figure S25. Subgroup analysis by hypertension. Figure S26. Subgroup analysis by age (older than 60). Figure S27. Subgroup analysis by age (including Spence's). Figure S28. Subgroup analysis by follow‐up duration. [file JAH3-6-e004180-s001.pdf]

Table S1. Detailed Definition of cataract and Adjustment Factors for cataract of observational studies

Study,year	Cataract definition	Adjustment
Schlienger, 2001[1]	Patients who had a first-time diagnosis of cataract(ICD-8)followed by a referral to a specialist or by a hospitalization because of cataract diagnosis, cataract surgery was identified by Oxford Medical Information System procedure code 156	age, sex, practice attended, calender time(by matching), BMI, smoking, number of general practitioner visit, corticosteroid use
Smeeth, 2003[2]	Patients who had a first-time diagnosis of cataract(ICD-8).	age, sex, observational period(by matching), smoking habit; body mass index; diabetes mellitus; glaucoma; hypertension; and exposure to aspirin, oestrogen (as hormone replacement therapy) and systemic corticosteroids, annual consultation rate
Fong, 2012[3]	Patient who had cataract extraction, identified with the CPT (current procedural terminology) code 66982, 66983,or 66984.	age, sex, ethnicity, coronary heart disease, diabetes, smoking
Wise-BC, 2014[4]	A cataract was defined as the first date of an ophthalmologist visit for cataract, and having a cataract surgical code within a year of the date of this visit	age, index date , follow-up years(by matching), sex (for the BC cohort), diabetes, hypertension, glaucoma, chronic obstructive pulmonary disease, uveitis, history of cardiovascular or cerebrovascular disease (previous history of stroke or myocardial infarction), and the following prescription drugs: antipsychotics, oral steroids, inhaled corticosteroids, and selective serotonin reuptake inhibitors
Wise-IMS, 2014[4]	A cataract was defined as the first date of an ophthalmologist visit for cataract, and having a cataract surgical code within a year of the date of this visit	age, index date , follow-up years(by matching), diabetes, hypertension, glaucoma, chronic obstructive pulmonary disease, uveitis, history of cardiovascular or cerebrovascular disease (previous history of stroke or

		myocardial infarction), and the following prescription drugs: antipsychotics, oral steroids, inhaled corticosteroids, and selective serotonin reuptake inhibitors
Erie, 2016[5]	Cataract and cataract surgery were retrospectively identified using the ICD-9 codes and CPT codes.	age,sex, diabetes, cardiovascular disease, cerebrovascular disease, peripheral vascular disease, renal disease, oral and inhaled steroid use, and selective serotonin reuptake inhibitor use.
Klein,2006[6]	Cataract was diagnosed by Wisconsin Cataract Grading System	age, sex, total cholesterol, high-density lipoprotein cholesterol, smoking, and diabetes
Tan, 2007[7]	Cataract was diagnosed by Wisconsin Cataract Grading System	sex, total cholesterol, high-density lipoprotein cholesterol, smoking, obesity, and diabetes
Cox, 2010[8]	Cataract was confirmed by patients' electronic records	age^3 , age^3 ln(age), ln(bmi), $bmi^{0.5}$, ethnicity, smoking, cardiovascular disease, type 1 diabetes, type 2 diabetes, rheumatoid arthritis, atrial fibrillation, corticosteroids; Cataract men: age^3 , age^3 ln(age), bmi^{-2} , bmi^{-1} , Townsend score, ethnicity, smoking, cardiovascular disease, type 1 diabetes, type 2 diabetes, atrial fibrillation, corticosteroids
Waudby, 2011[9]	Cataract and cataract surgery were identified using the ICD-9 codes and CPT codes.	sex, diabetes, smoking, steroid use, BMI, HDL, antioxidant
Lai, 2013[10]	Cataract surgery was identified by ICD-9 codes	age, sex, diabetes mellitus under treatment, hypertension undertreatment, Charlson Index such as

		myocardial infarction, cerebrovascular disease, chronic pulmonary disease, renal disease, liver disease and malignancy, oral estrogen replacement therapy, corticosteroids, non-steroidal anti-inflammatory drugs, aspirin, proton pump inhibitors, H2 receptor antagonists, number of distinct prescription drugs dispensed, number of hospitalizations and number of physician visits were also included in the list of potential confounders.
Leuschen, 2014[11]	Cataracts were difined using ICD-9 codes	age, sex, obesity, smoking, alcohol use, illicit drug use, glaucoma at baseline, vision defects/blindness, number of all admissions during baseline, number of all outpatient visits during baseline, and use of different classes of medications as listed in Table 1. mean low-density lipoprotein cholesterol.

Table S2. NOS for Assessment of Quality of Included Studies: Case-Control Studies

Quality Assessment criteria	Acceptable	Schlienger 2001[1]	Smeeth 2003[2]	Fong 2012[3]	Wise-BC 2014[4]	Wise-IMS 2014[4]	Erie 2016[5]
Selection<br>Is the case definition adequate?	Yes, with independent validation	–	–	–	–	–	–
Repsentiveness of cases?	Consecutive or obviously representative series of cases	+	+	–	–	+	–
Selection of controls?	Community controls	+	+	+	+	+	+
Definition of controls?	No history of cataracts	+	+	+	+	+	+
Comparability<br>Study controls for age/sex	Yes	+	+	+	+	+	+
Study controls for any other confounding factors?	Yes	-	–	–	–	–	–
Exposure<br>Ascertain of exposure?	Secure record, Structured interview where blind to case-control status	+	+	+	+	+	+
Same method of ascertainment of cases/controls?	Yes	+	+	+	+	+	+
Nonresponse rate	Same for both the groups	+	+	+	+	+	+
Overall quality score (maximum=10)		7	7	6	6	7	6

Table S3. NOS for Assessment of Quality of Included Studies: Cohort Studies

Quality assessment criteria	Acceptable	Klein 2006[6]	Tan 2007[7]	Cox 2010[8]	Waudby 2011[9]	Lai 2013[10]	Leuschen 2014[11]
Selection<br>Representativeness of exposed cohort?	Representative of average adult in community (age/sex/being at risk of disease)	+	–	+	+	+	-
Selection of the nonexposed cohort?	Drawn from same community as exposed cohort	+	+	+	+	+	+
Ascertainment of exposure?	Secured records, structured interview	+	+	+	+	+	+
Demonstration that outcome of interest was not present at the start of the study?	Only incident cases of cataracts	+	+	+	+	+	+
Comparability<br>Study controls for age/sex	Yes	+	+	+	+	+	+
Study controls for any other confounding factors?	Yes	–	–	–	–	–	–
Exposure<br>Assessment of outcomes?	Independent blind assessment record linkage	+	+	+	+	+	+
Was follow-up evaluation long enough for outcome to occur?	Yes	+	+	+	+	+	+
Adequacy of follow-up evaluation of cohorts	Complete follow-up evaluation, or subjects lost to follow-up evaluation unlikely to introduce bias	–	–	+	–	+	+
Overall quality score(maximum=10)		7	6	8	6	8	7

Table S4. Quality of the included RCTs assessed by Cochrane risk of bias assessment

Quality assessment criteria	Laties 1991[12]	Harris 1995[13]	Pederson 1996[14]	Bang 2015[15]	Heart Protection Study 2015[16]
Random sequence generation	-	+	+	-	+
Allocation concealment	+	+	+	-	+
Blinding participants and personnel	+	-	+	+	+
Blinding of outcome assessment	+	-	-	-	-
Incomplete outcome data	-	+	+	+	+
Selective reporting	+	+	+	+	+
Other bias	+	+	+	+	+
Overall quality score (maximum=7)	5	5	6	5	6

Table S5. Subgroup analysis of cohort studies

Factor	Studies n	RR (95% CI)	I^2
Quality assessment			
High (≥7)	4	1.15(1.02-1.30)	92.8%
Low (<7)	2	0.83 (0.37-1.83)	56.4%
Study location			
North America	3	1.05 (0.89-1.24)	78.0%
Europe	1		
Asia	1		
Australia	1		
Sex			
Male	3	1.24 (1.12-1.37)	64.0%
Female	2	1.30 (1.25-1.34)	0%
Outcome assessment			
ICD codes	3	1.20 (1.14-1.25)	0%
Wisconsin Cataract Grading System	2	0.57 (0.40-0.81)	0%
Outcome			
Cataract	4	1.17 (1.03-1.34)	82.6%
Cataract surgery	1		
Cataract and surgery	1		
Age			
Older than 60 years	4	1.00 (0.82-1.22)	82.6%
No older than 60 years	2	1.28 (1.19-1.38)	48.7%
Follow-up duration			
Less than 5 years	2	1.28(1.19-1.38)	48.7%
5 or more than 5 years	4	1.00(0.82-1.22)	82.6%

Factor	Studies n	RR (95% CI)	I^2
Whether LDL included			
LDL included model	3	0.75 (0.41-1.37)	87.3%
LDL missing model	3	1.24 (1.15-1.34)	82.1%
Whether CVD included			
CVD included model	2	1.26(1.15-1.37)	88.4%
CVD missing model	4	0.96(0.75-1.22)	81.7%
Whether smoking included			
Smoking included model	5	1.13 (0.98-1.30)	82.1%
Smoking missing model	1		
Whether consultation rate included			
consultation rate included model	2	1.20(1.14-1.26)	0%
consultation rate missing model	4	1.01(0.81-1.27)	88.0%
Whether hypertension included			
Hypertension included model	2	1.26 (1.15-1.37)	88.4%
Hypertension missing model	4	1.01(0.82-1.25)	76.2%

Table S6. Subgroup analysis of case-control studies

Factor	Studies n	RR (95% CI)	I^2
Quality assessment			
High (≥7)	3	1.07(1.04-1.10)	0%
Low (<7)	3	1.15 (0.96-1.37)	95.9%
Study location			
North America	4	1.13 (0.99-1.28)	97.5%
Europe	2	1.02 (0.90-1.16)	0%
Outcome assessment			
ICD codes	3	1.12 (0.94-1.33)	77.1%
Medical records	2	1.17 (0.99-1.38)	98.8%
Type of statins			
Artovastatin	4	1.17 (1.04-1.31)	90.0%
Fluvastatin	3	1.23 (0.96-1.56)	53.1%
Lorvastatin	3	1.22 (1.07-1.39)	74.1%
Pravastatin	3	1.23 (0.96-1.56)	53.1%
Simvastain	4	1.13 (0.99-1.30)	92.9%
Rosuvastatin	2	1.21 (0.89-1.65)	94.2%
Outcome			
Cataract surgery	3	1.16 (0.95-1.42)	86.8%
Cataract and surgery	2	1.13(0.97-1.31)	97.7%
Cataract	1	1.04(0.89-1.23)	
Whether CVD included			
CVD included model	4	1.17(1.04-1.31)	96.7%
CVD missing model	2	1.02(0.92-1.18)	0%

Factor	Studies n	RR (95% CI)	I^2
Whether smoking included			
Smoking included model	3	1.03 (0.96-1.13)	0%
Smoking missing model	3	1.12 (1.05-1.37)	97.7%
Whether consultation rate included			
consultation rate included model	2	1.02(0.90-1.16)	0%
consultation rate missing model	4	1.17(1.04-1.31)	96.7%
Whether diabetes included			
Diabetes included model	5	1.15 (1.03-1.27)	95.8%
Diabetes missing model	1	1.00(0.80-1.20)	
Whether hypertension included			
Hypertension included model	4	1.17 (1.04-1.32)	96.7%
Hypertension missing model	2	1.03 (0.93-1.15)	0%

Table S7. Subgroup analysis of RCTs

Factor	Studies n	RR	I^2
Age			
Older than 60 years	3	0.82 (0.53-1.26)	52.2%
Young than 60 years	1	0.89 (0.39-2.07)	
Follow-up duration			
More than 5 years	2	0.95 (0.83-1.08)	0%
Less than 5 years	3	0.86 (0.48-1.51)	53%

Figure S1. Sensitivity analysis of cohort studies

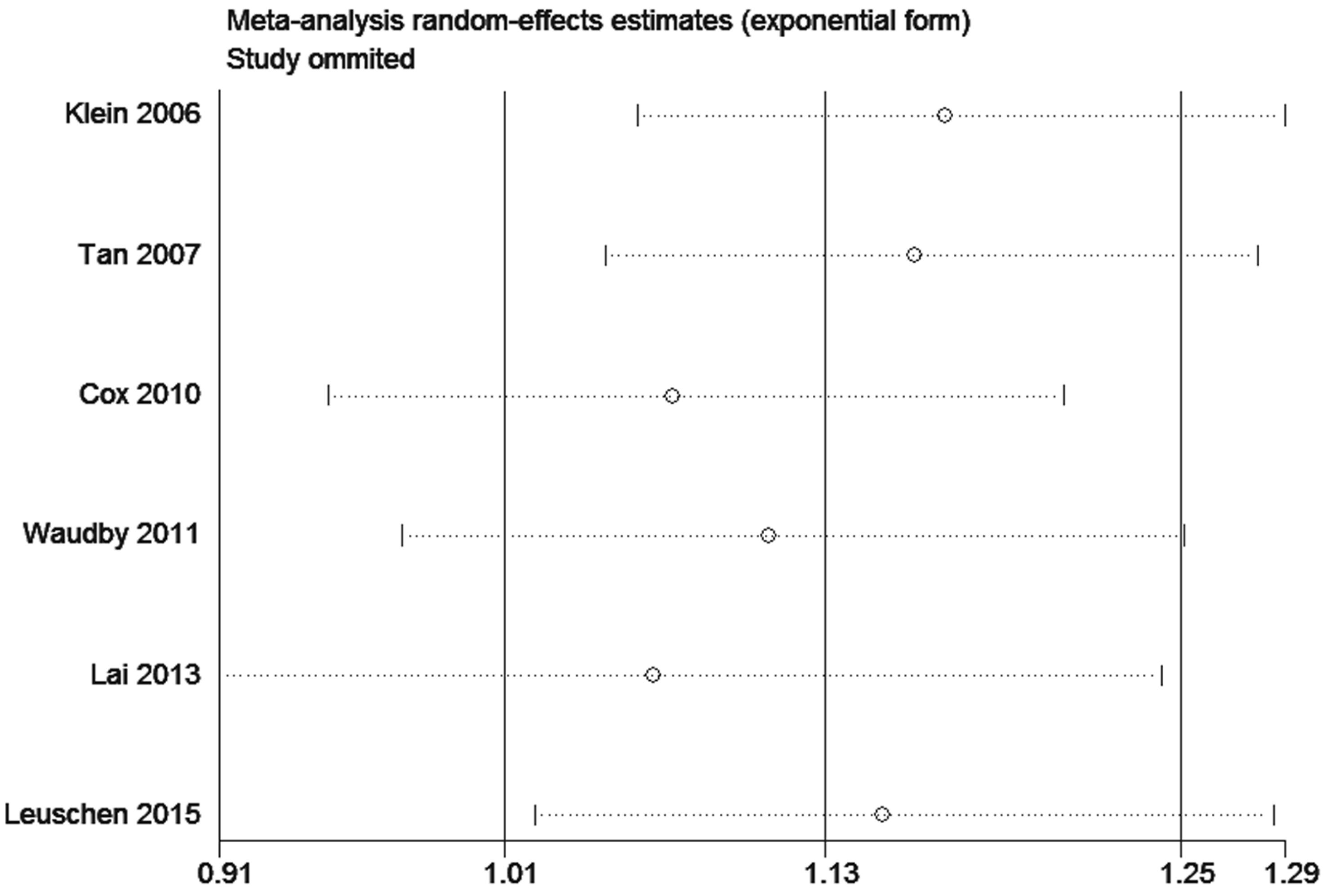

Figure S2. Sensitivity analysis of case-control studies

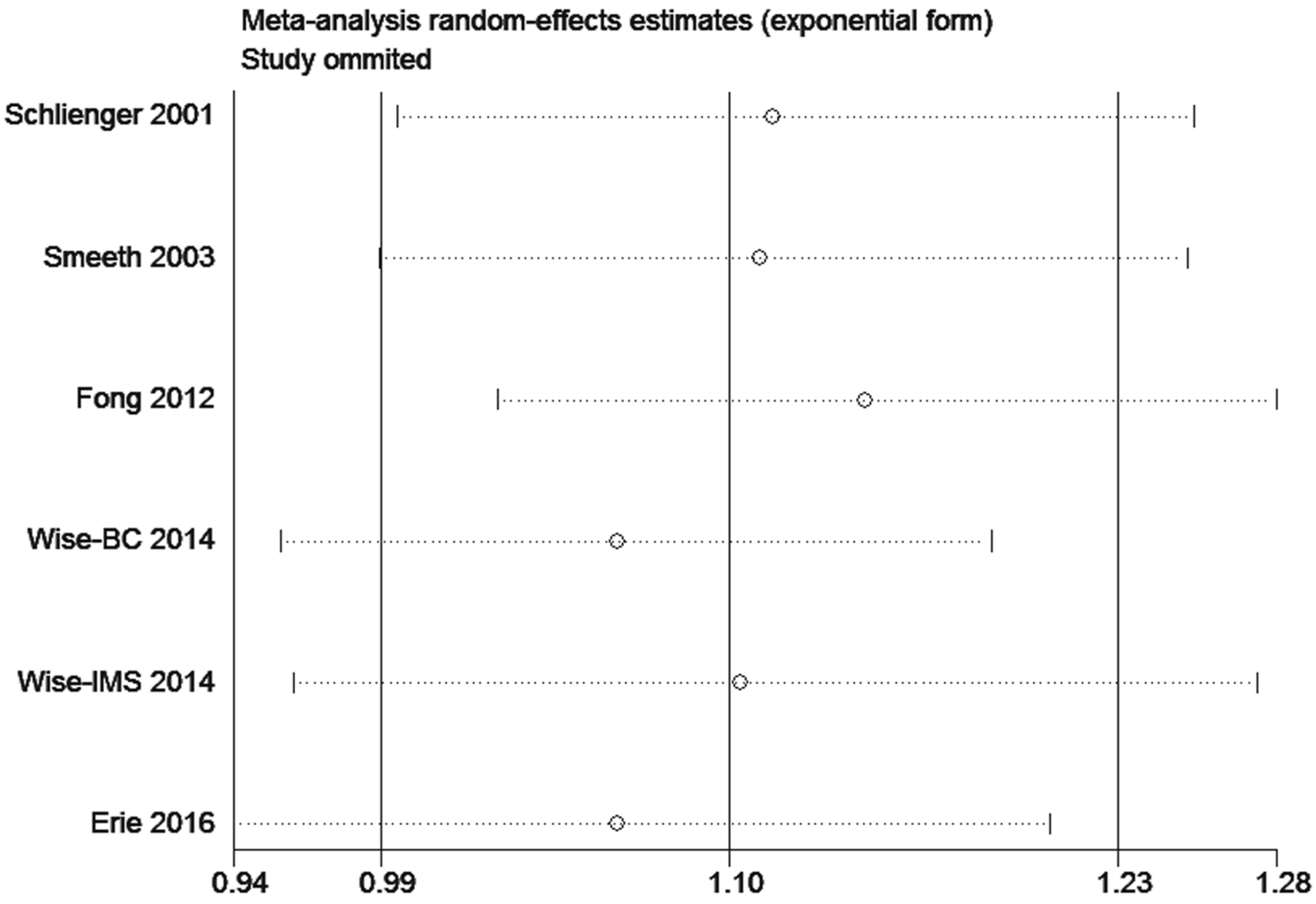

Figure S3. Sensitivity analysis of RCTs

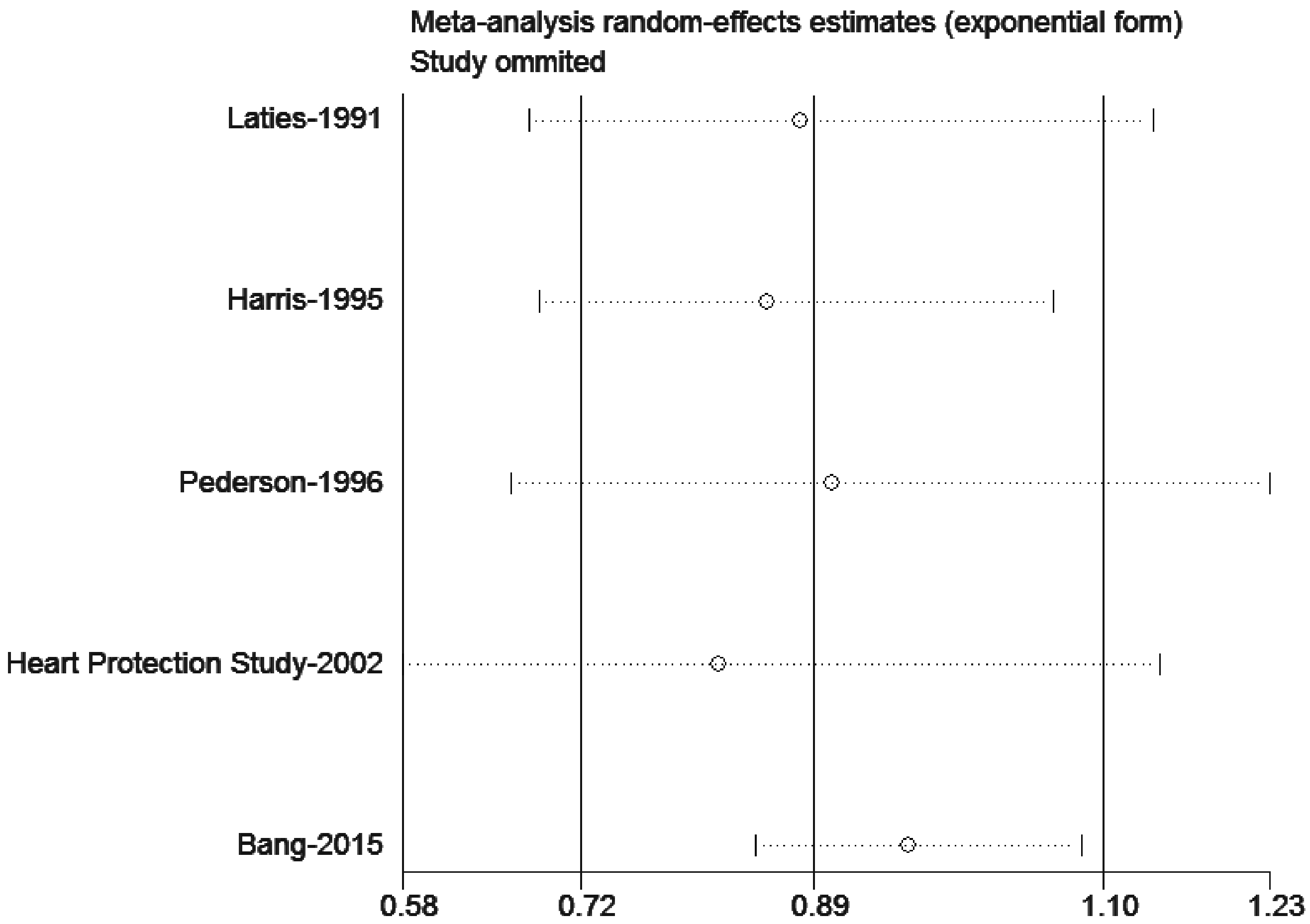

Figure S4. Subgroup analysis of cohort by quality assessment

A. High quality

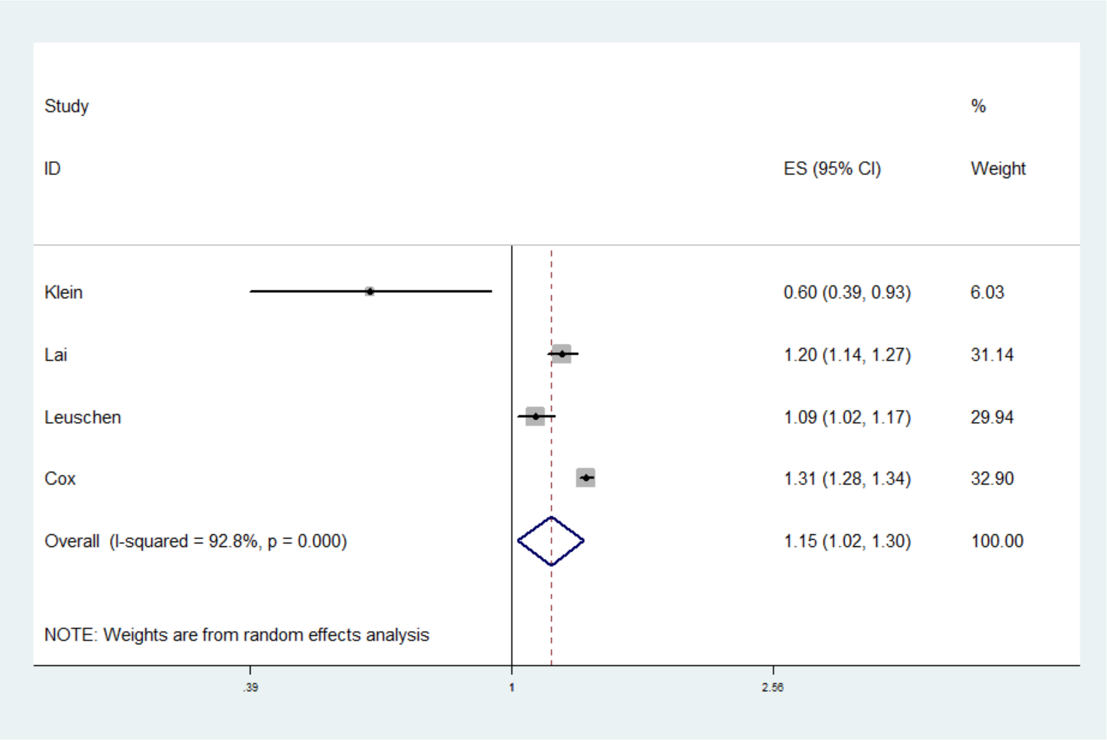

B. Low quality

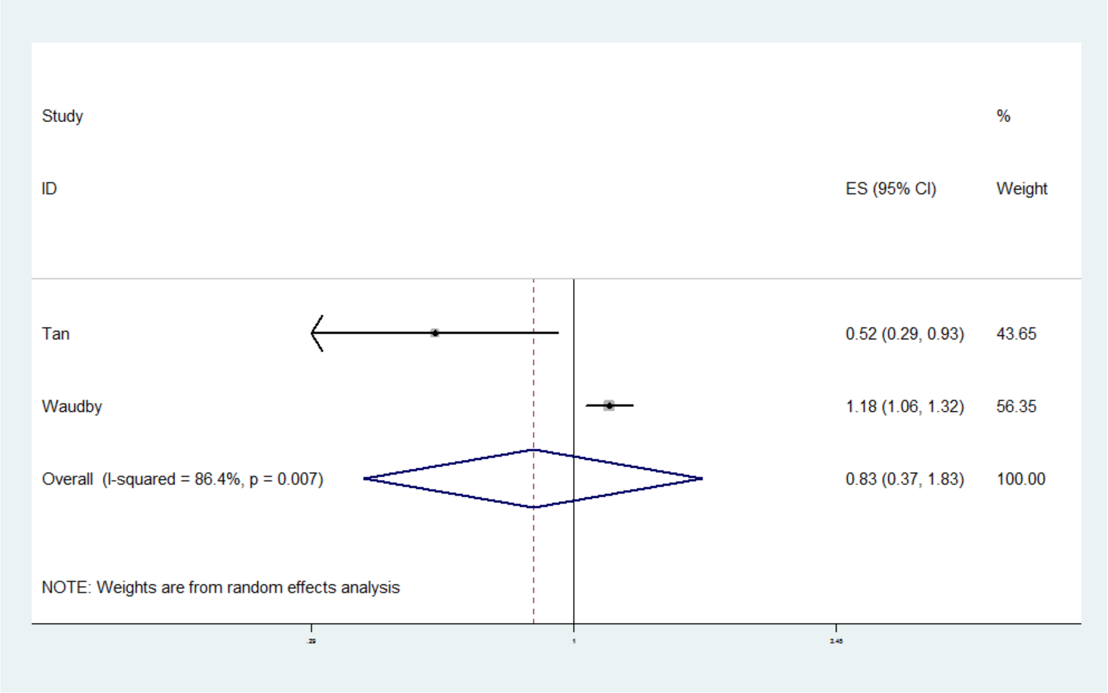

Figure S5. Subgroup analysis of cohort by location (North America)

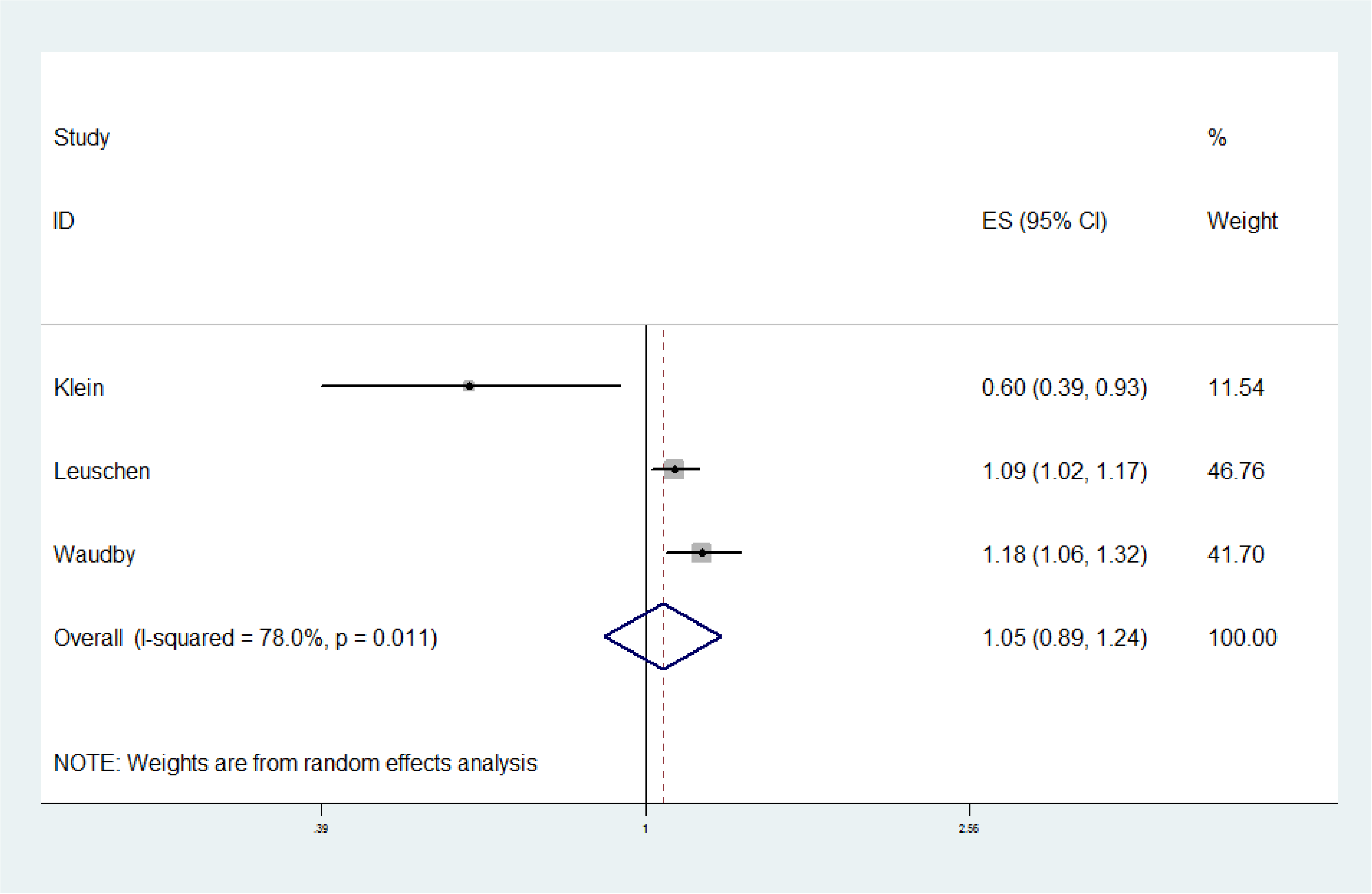

Figure S6. Subgroup analysis of cohort by sex

A. Male

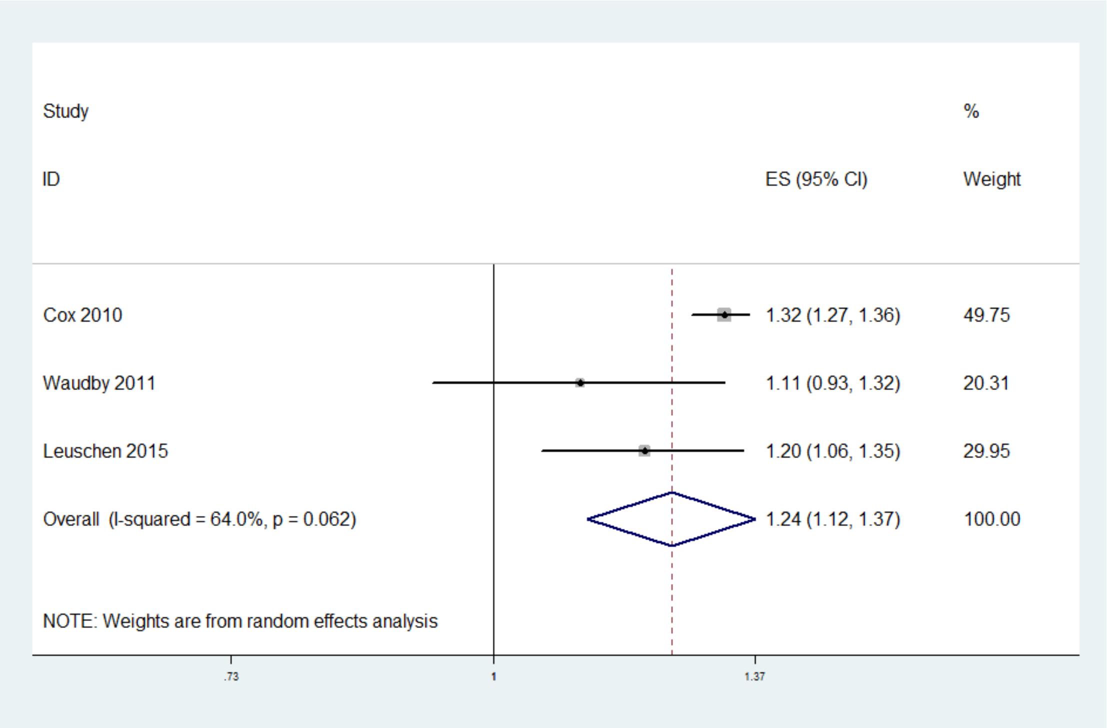

B. Female

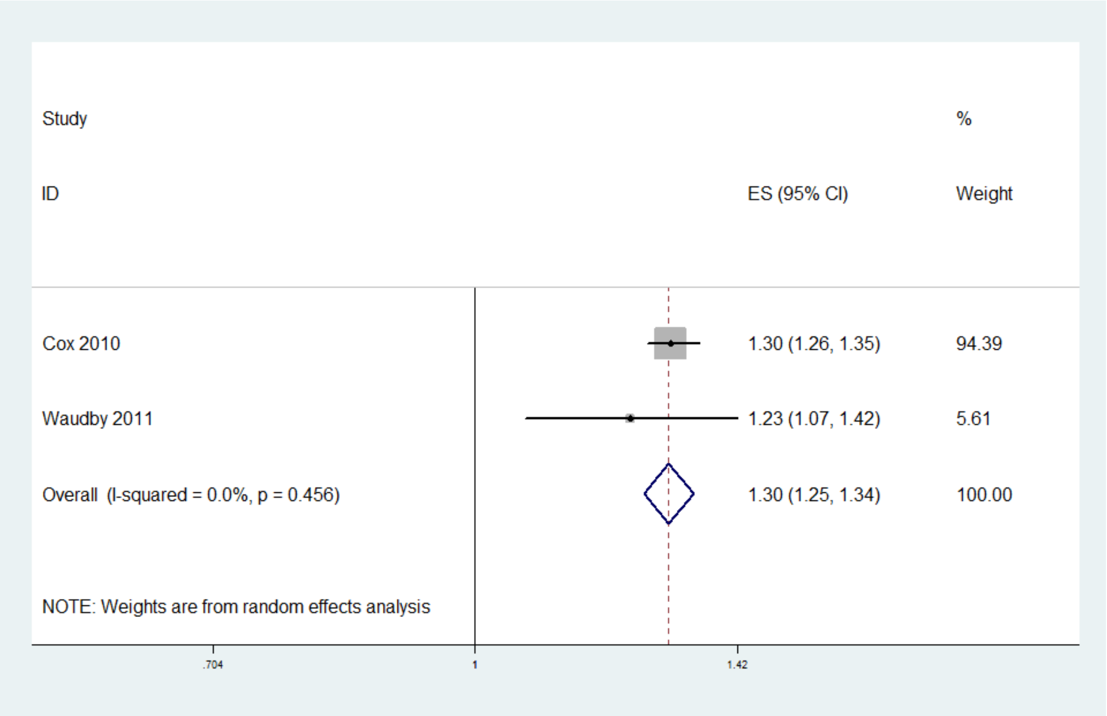

Figure S7. Subgroup analysis of cohort by outcome assessment

A. ICD codes

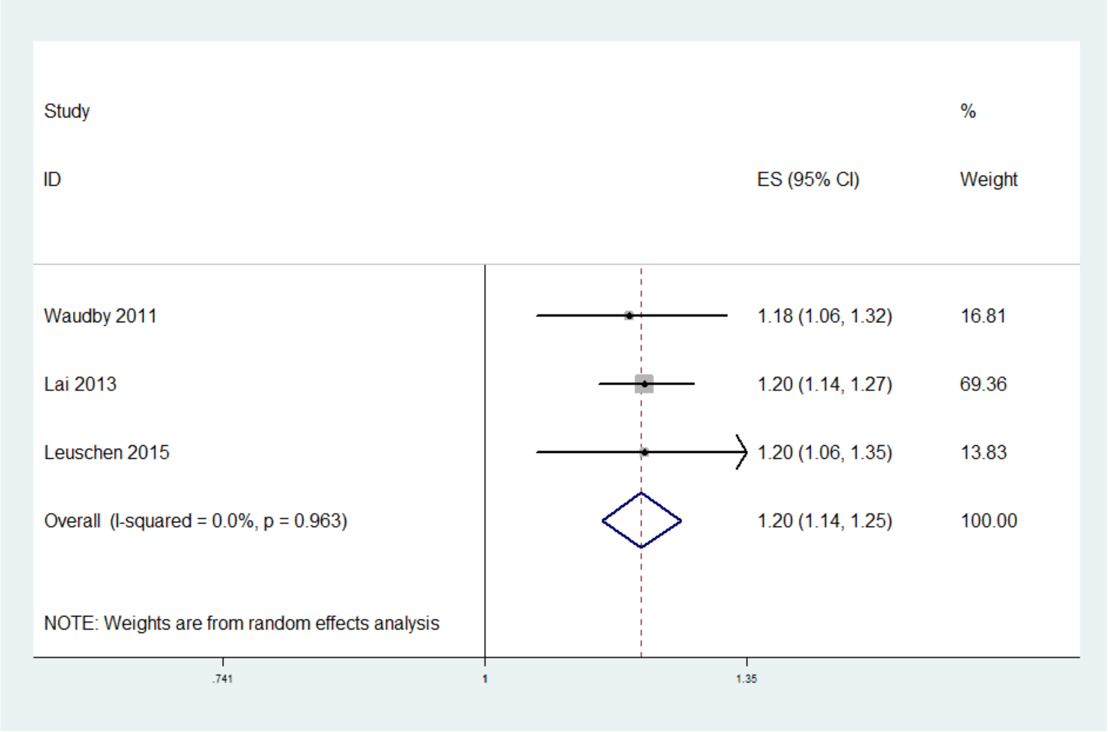

B. Grading system

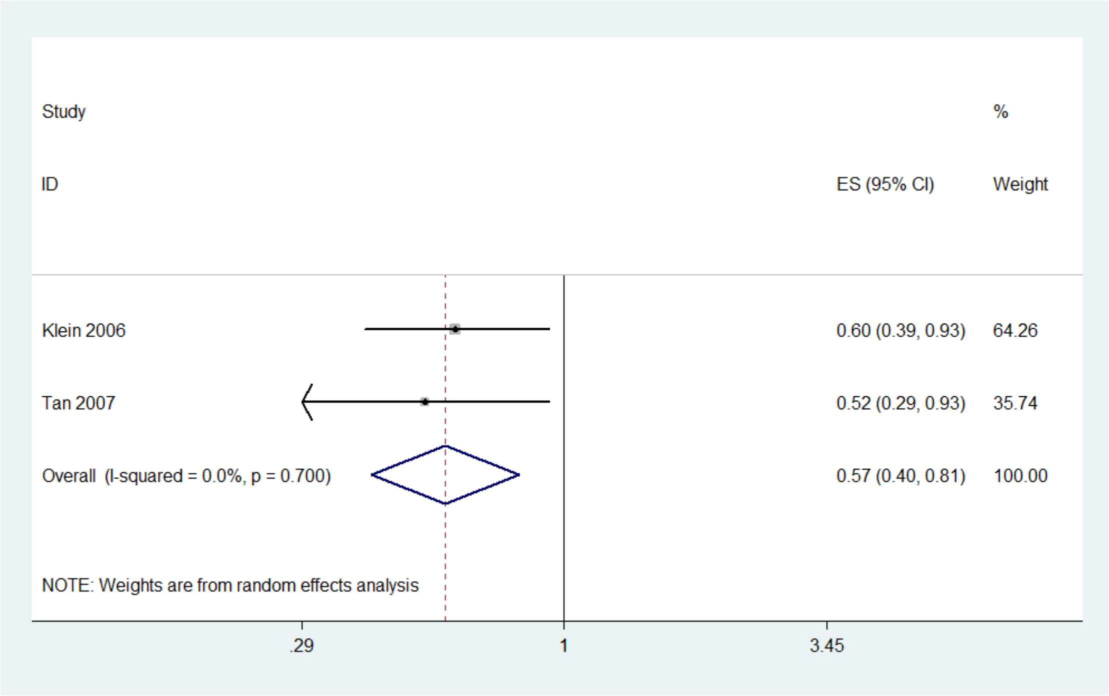

Figure S8. Subgroup analysis of cohort by outcome (Cataract)

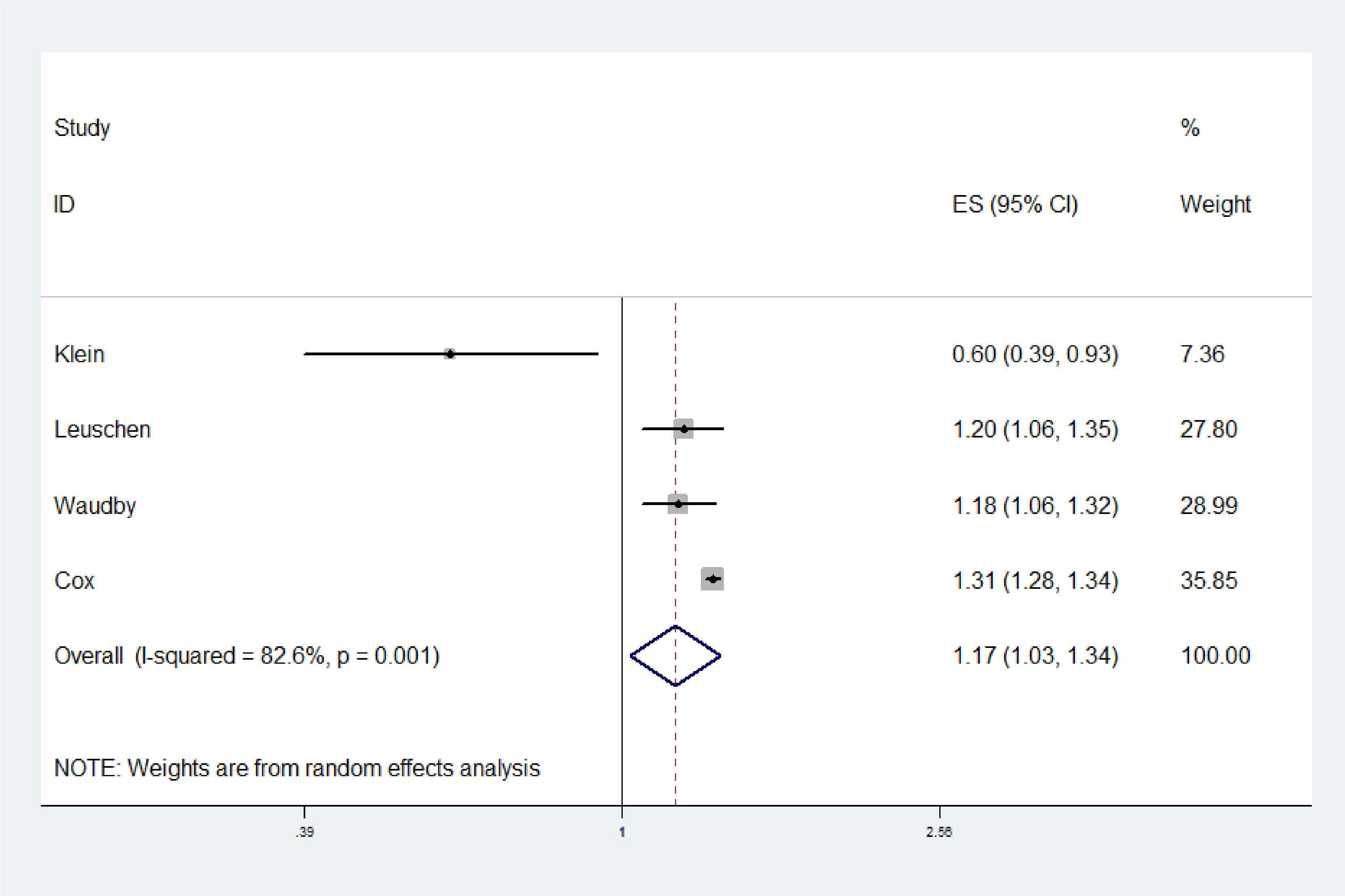

Figure S9. Subgroup analysis of cohort by age

A. Older than 60

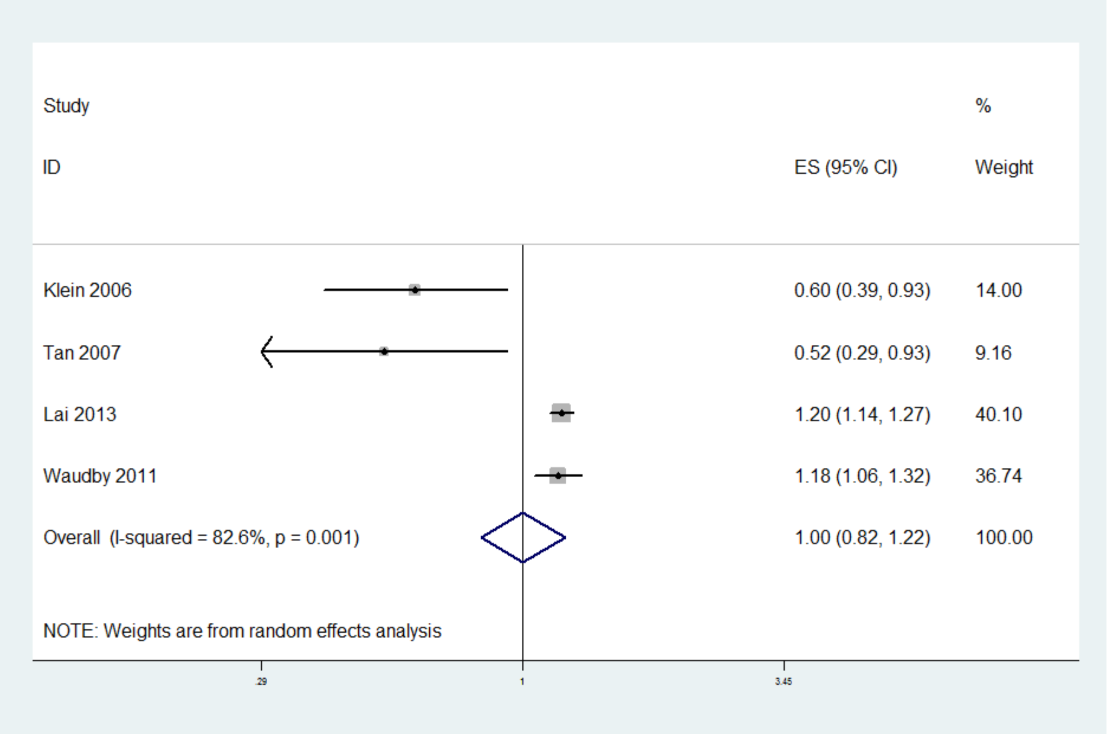

B. Younger than 60

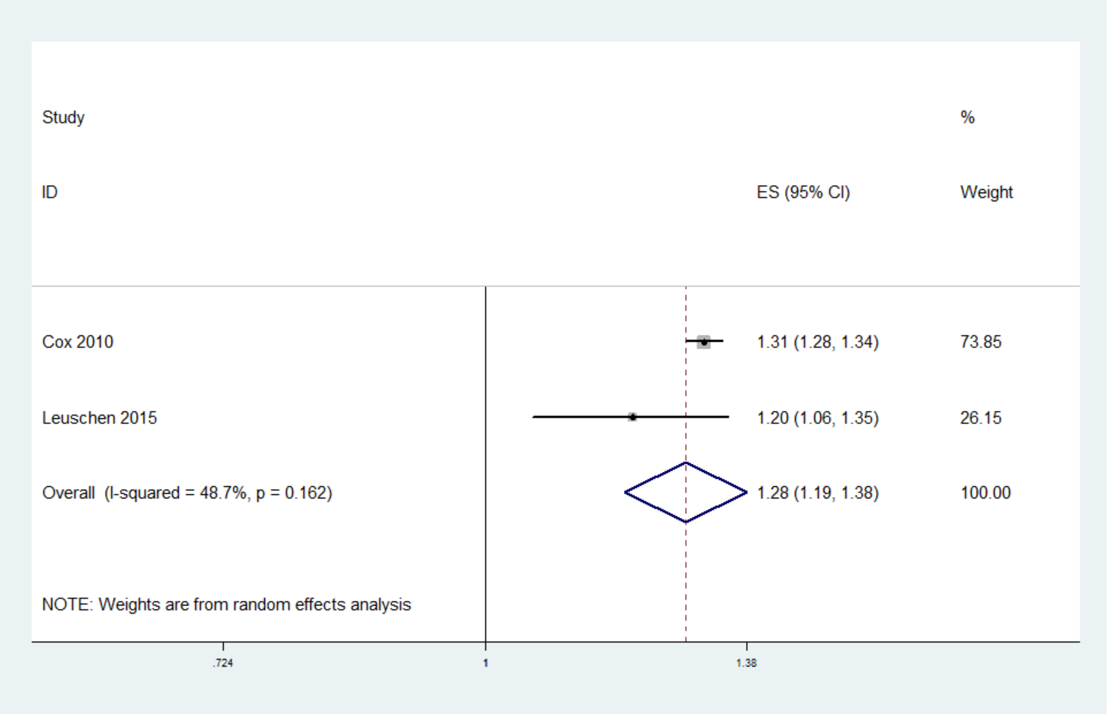

Figure S10. Subgroup analysis of cohort by follow-up duration

A. More than 5 years

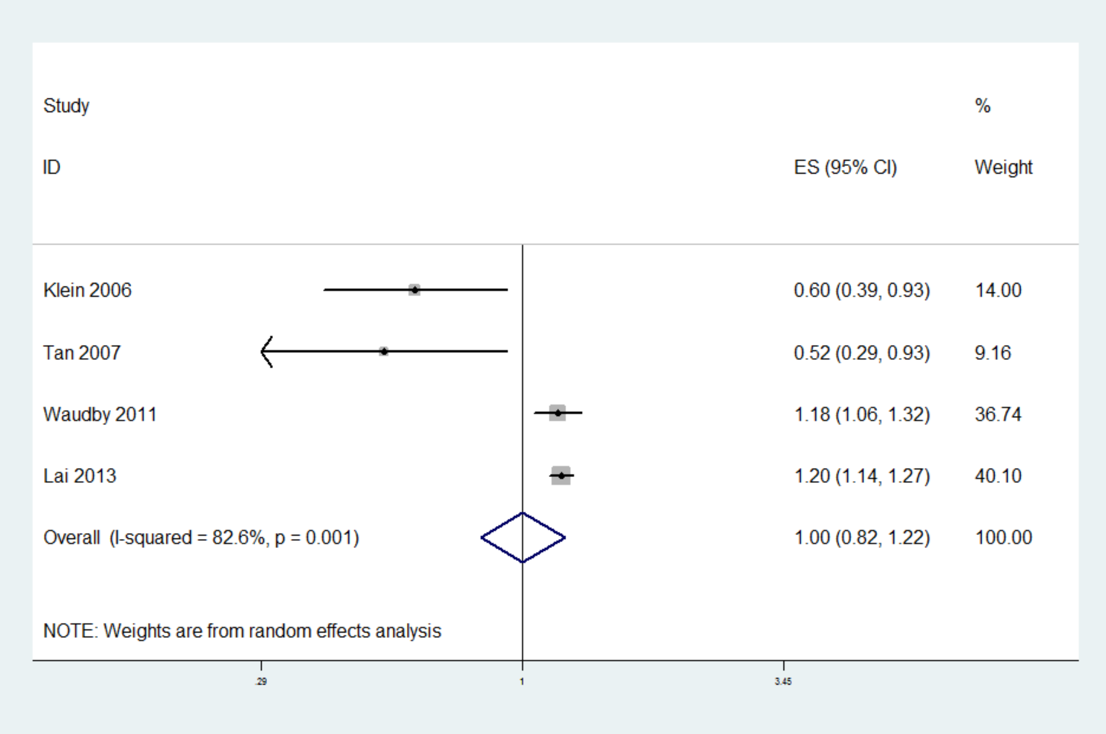

B. Less than 5 years

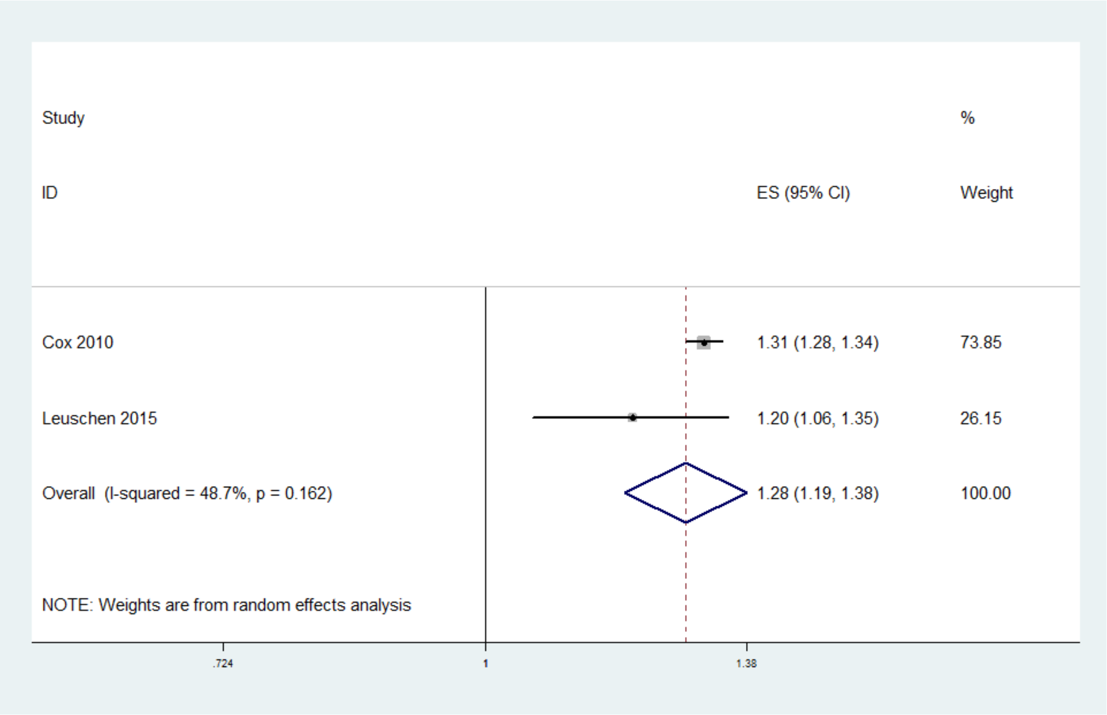

Figure S11. Subgroup analysis of cohort by whether LDL included

A. LDL included

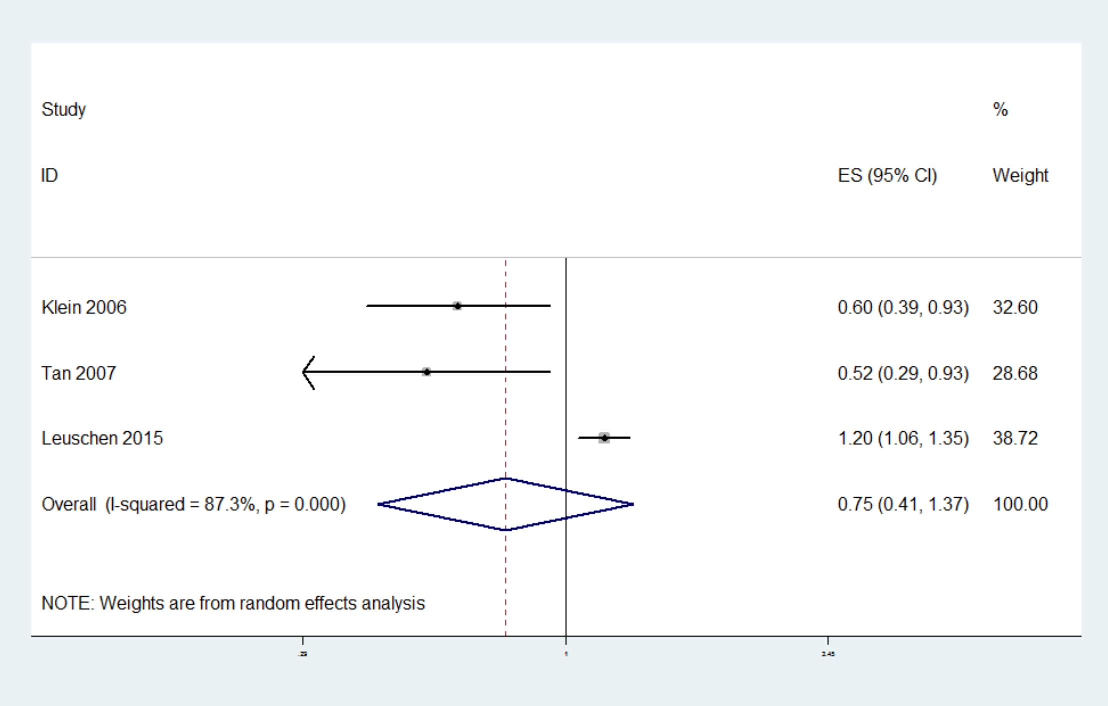

B. LDL missing

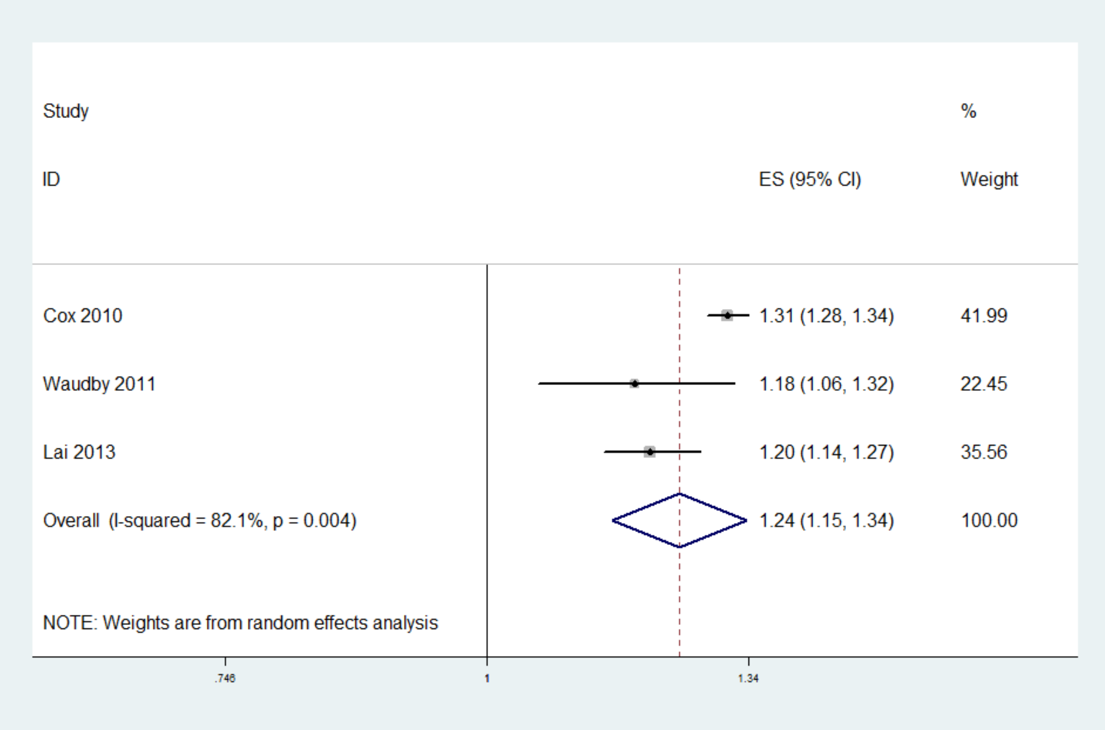

Figure S12. Subgroup analysis of cohort by CVD

A. CVD included

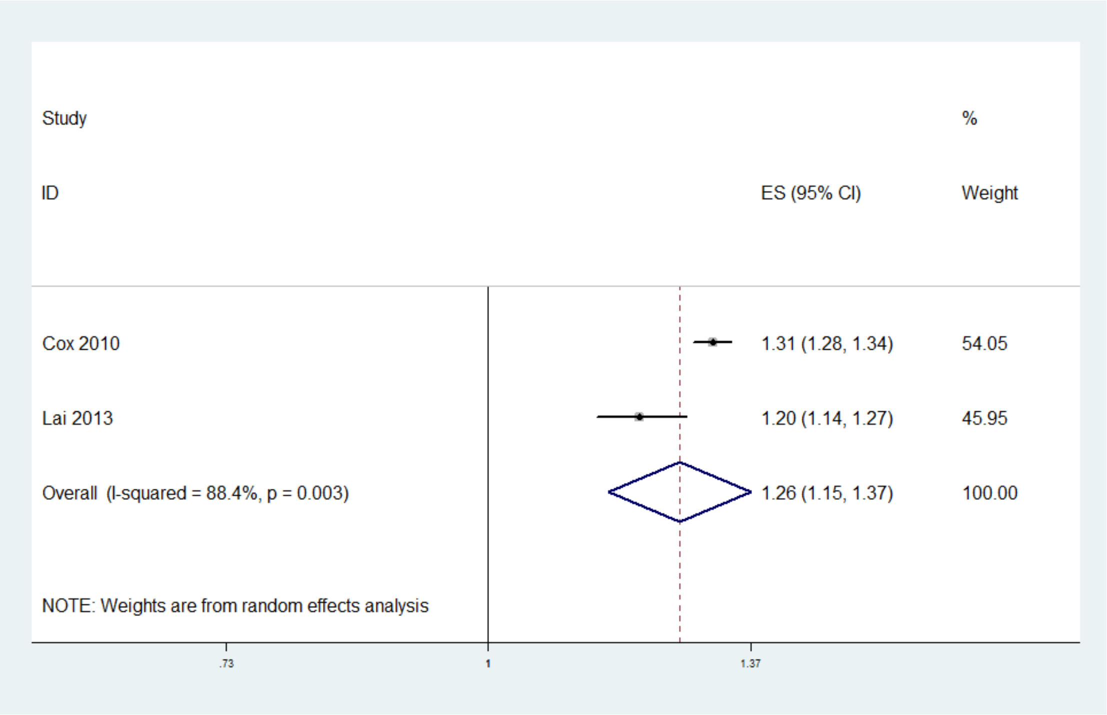

B. CVD missing

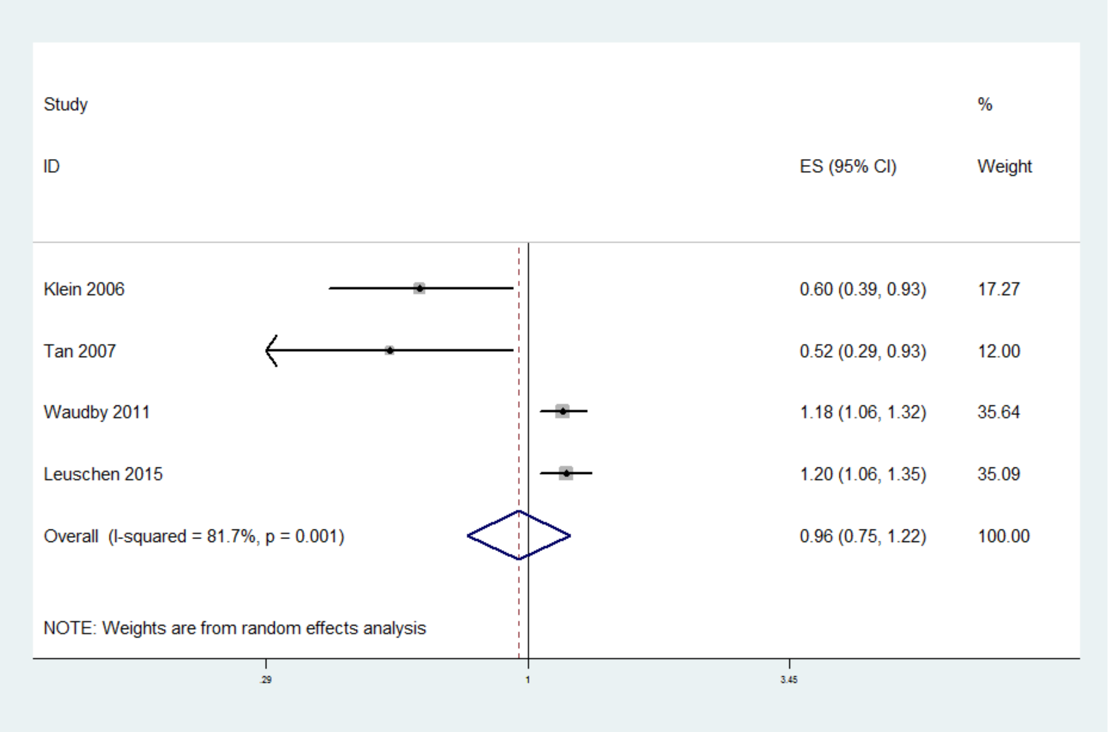

Figure S13. Subgroup analysis of cohort by smoking (Smoking included)

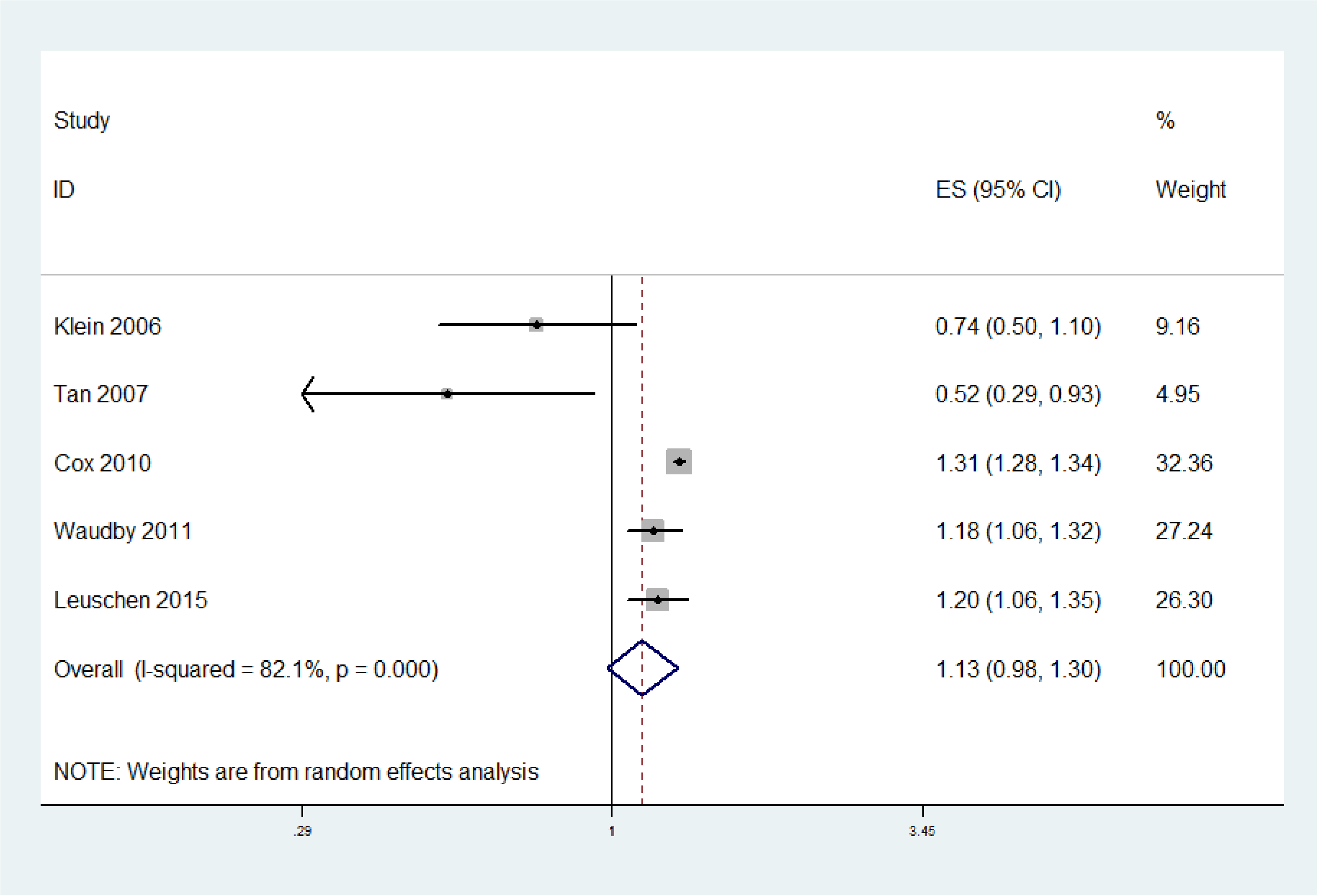

Figure S14. Subgroup analysis of cohort by consultation rate

A. Consultation rate included

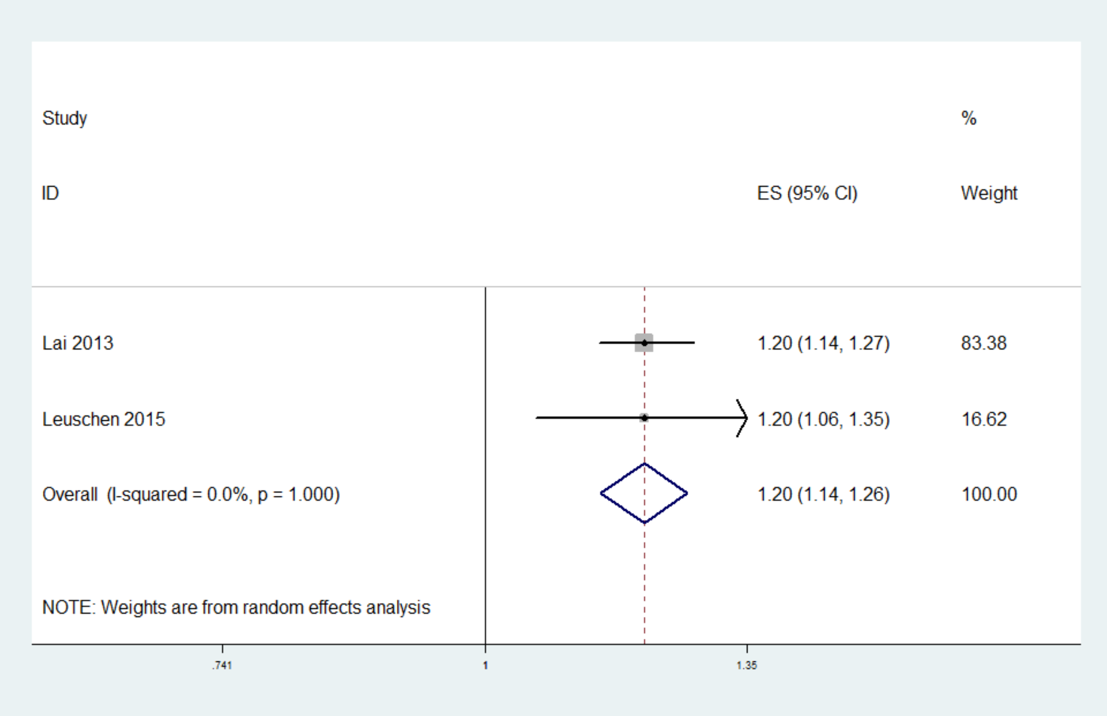

B. Consultation rate missing

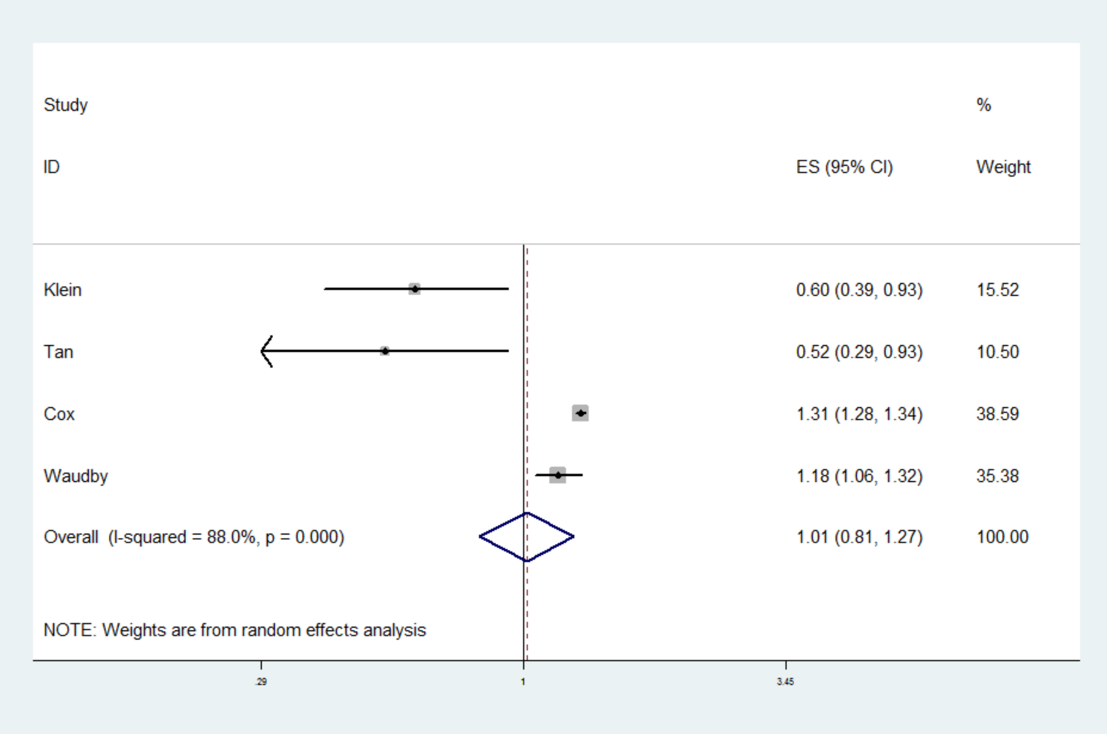

Figure S15. Subgroup analysis of cohort by hypertension

A. Hypertension included

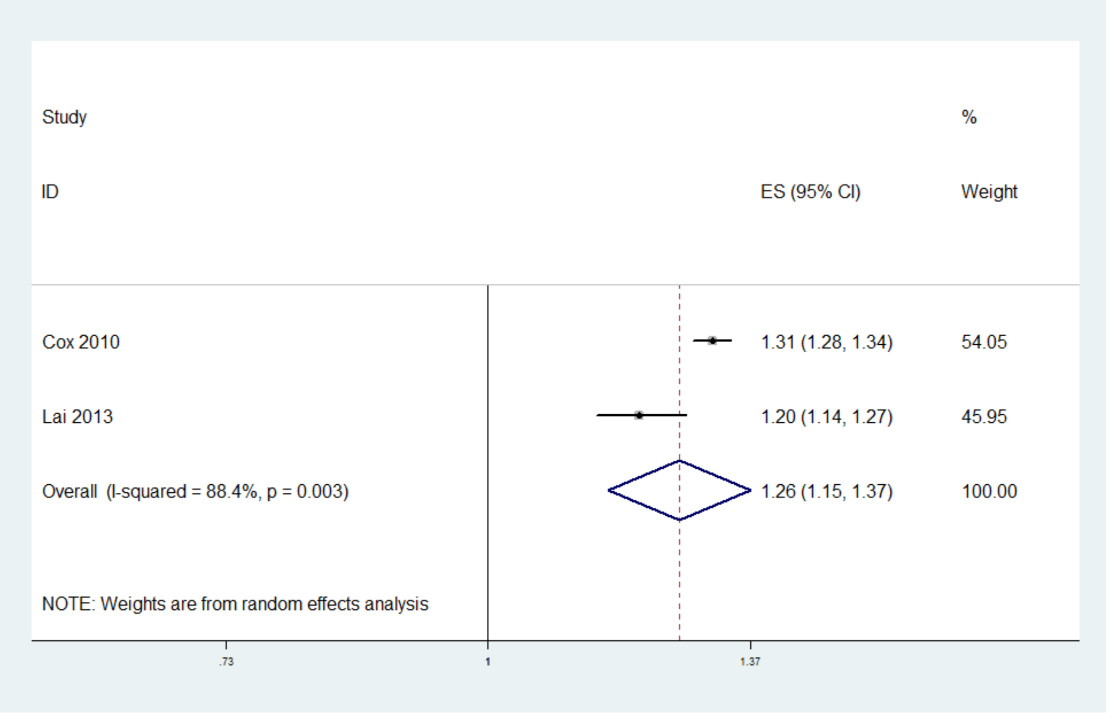

B. Hypertension missing

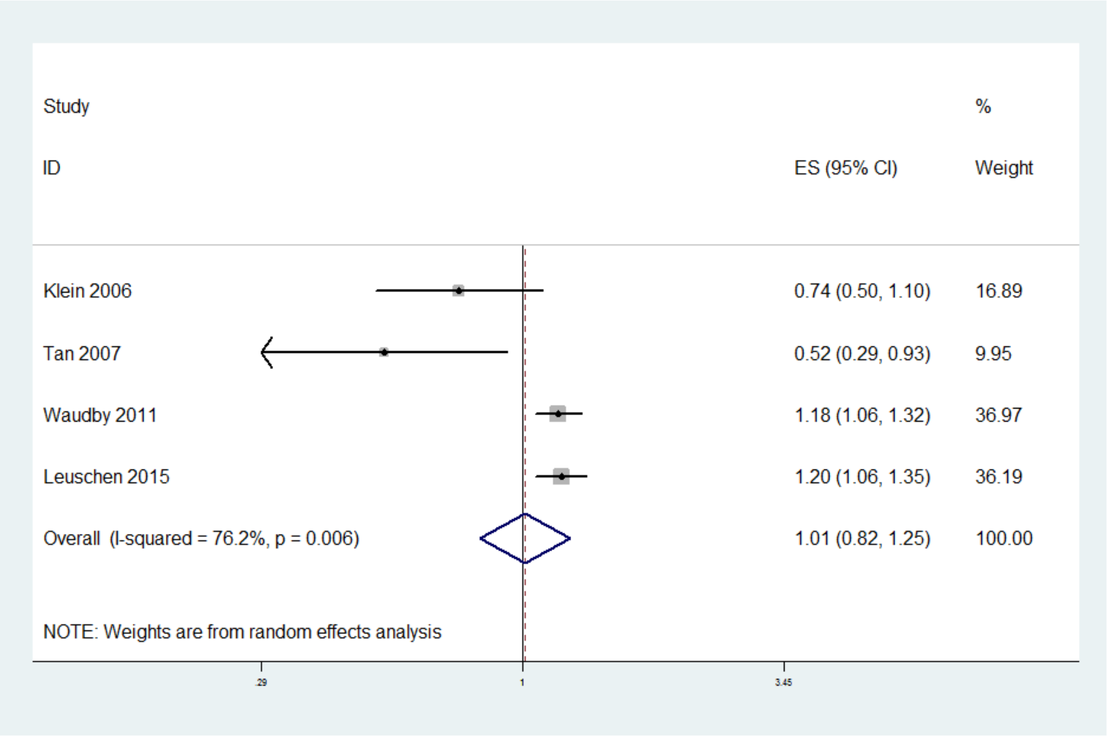

Figure S16. Subgroup analysis of case-control by quality assessment

A. High quality

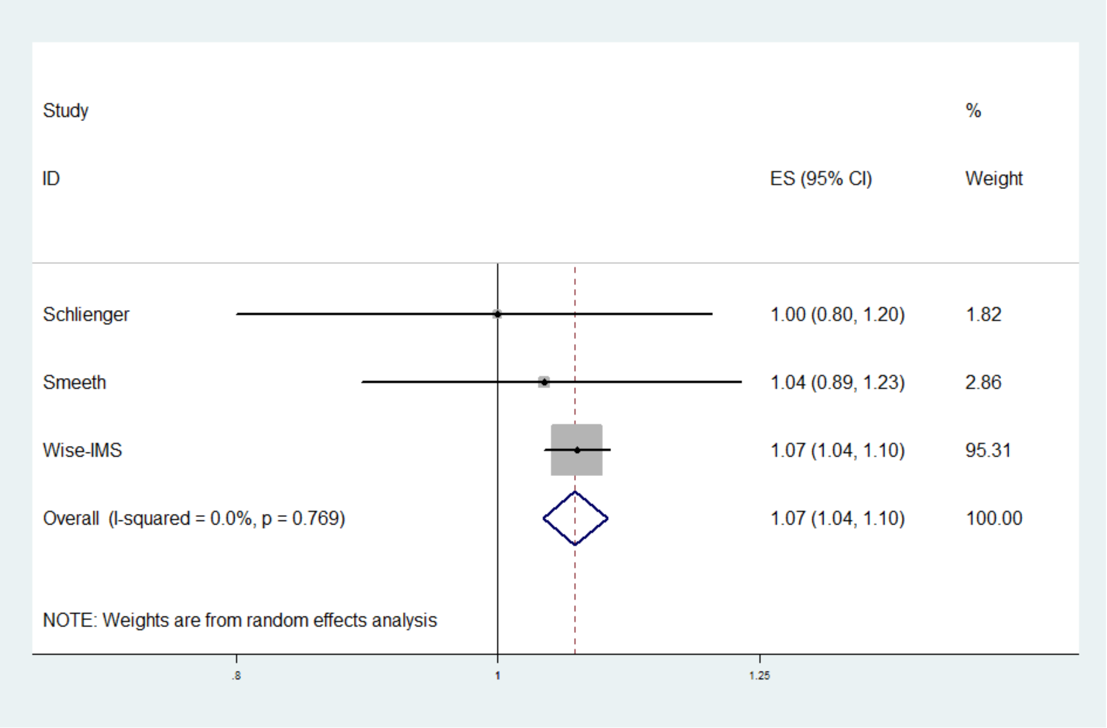

B. Low quality

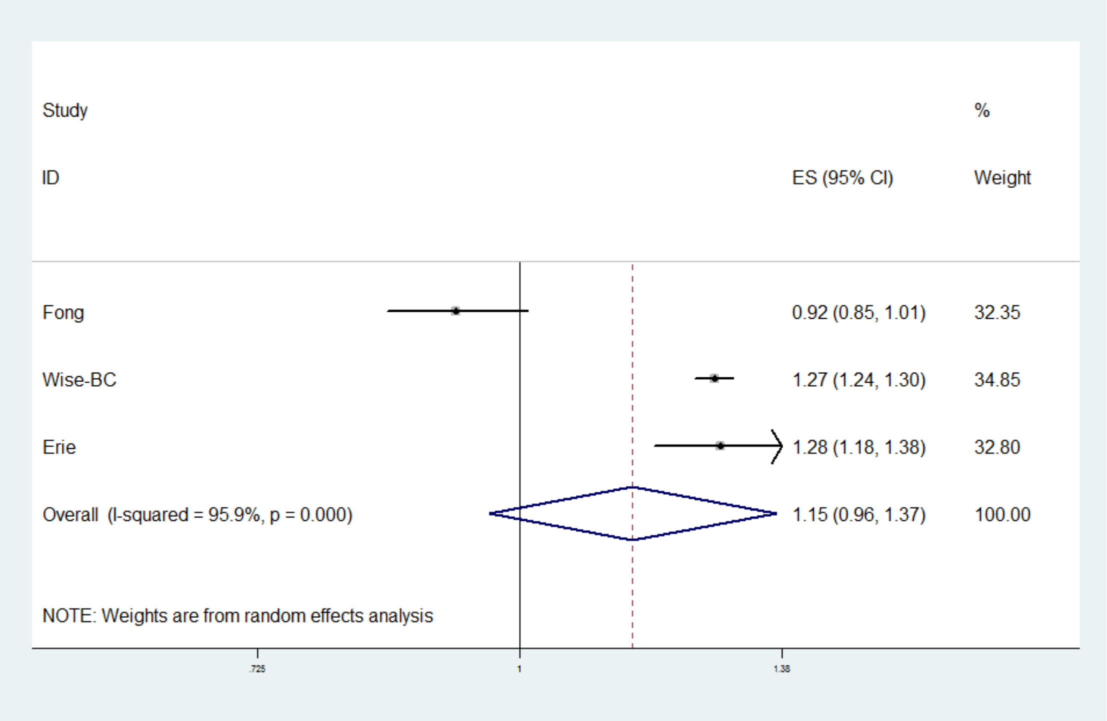

Figure S17. Subgroup analysis of case-control by location

A. North America

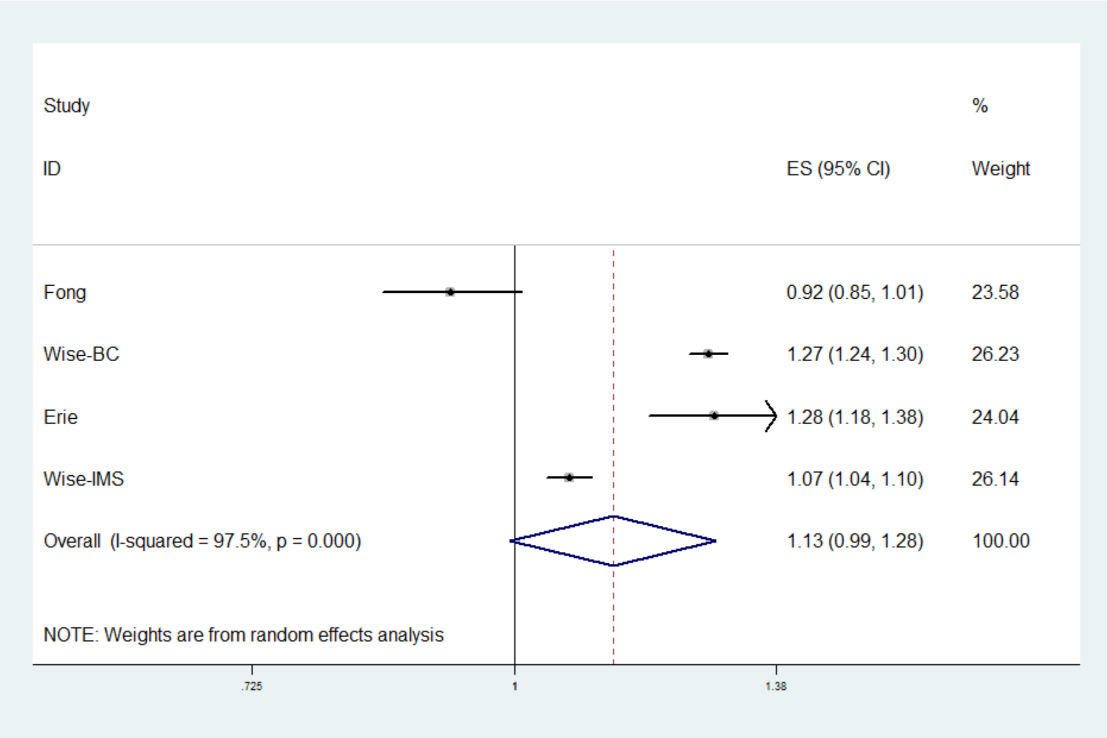

B. Europe

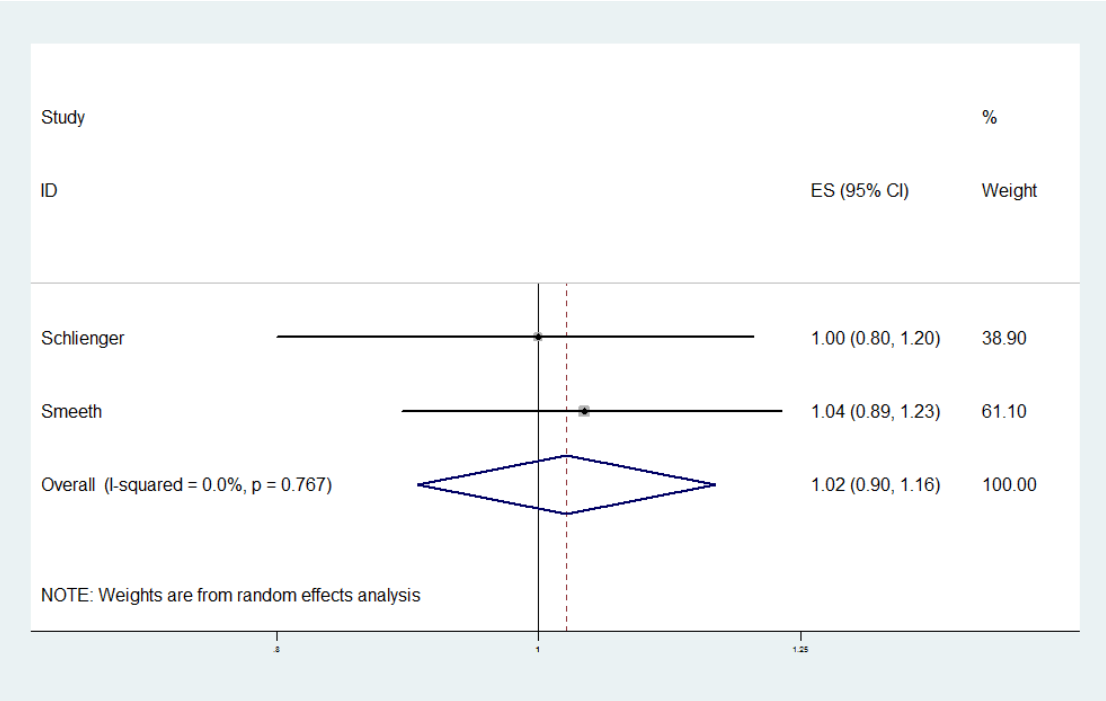

Figure S18. Subgroup analysis of case-control by outcome assessment

A. ICD codes

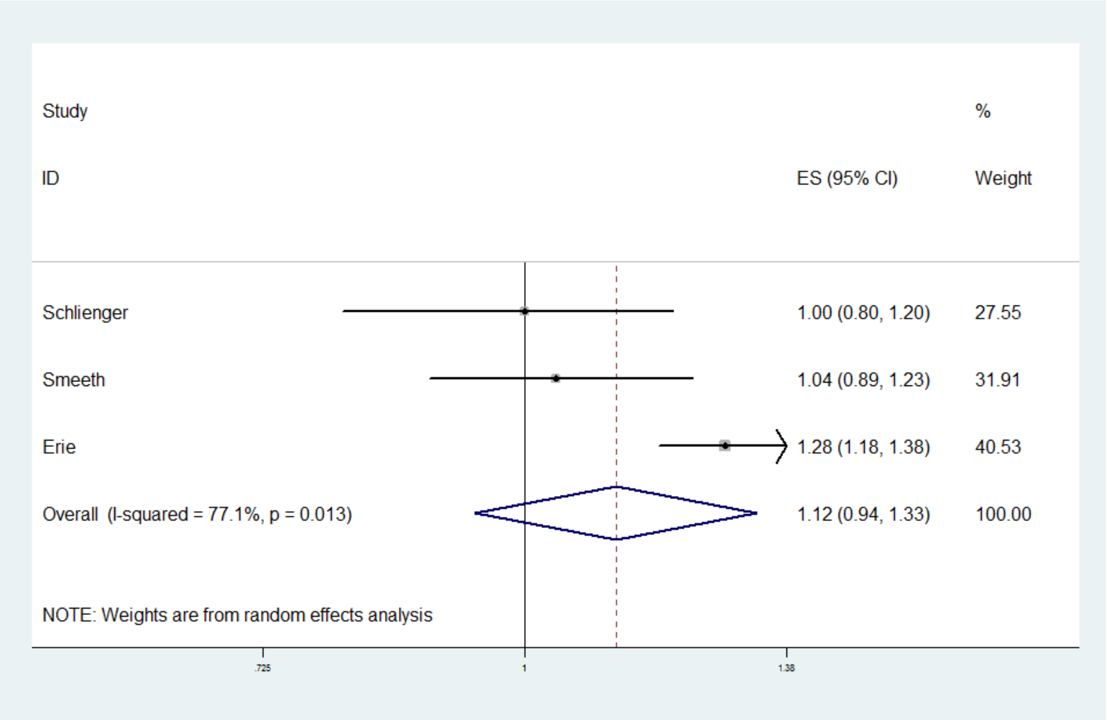

B. Medical records

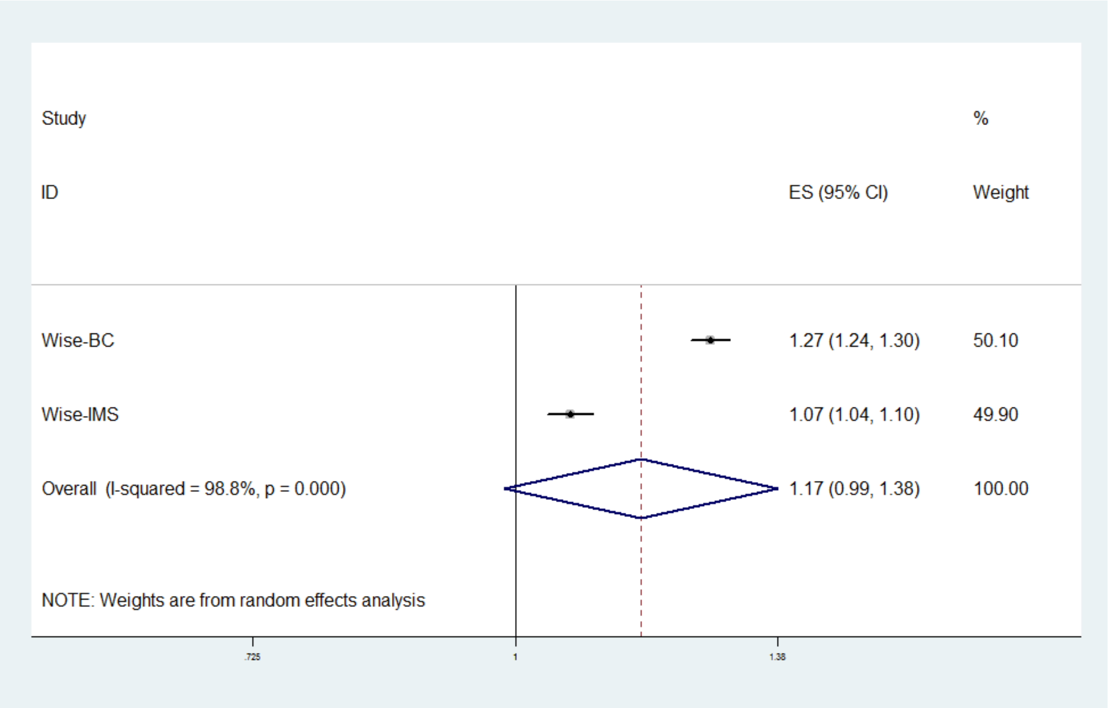

Figure S19. Subgroup analysis of case-control by type of statins

A. Artovastatin

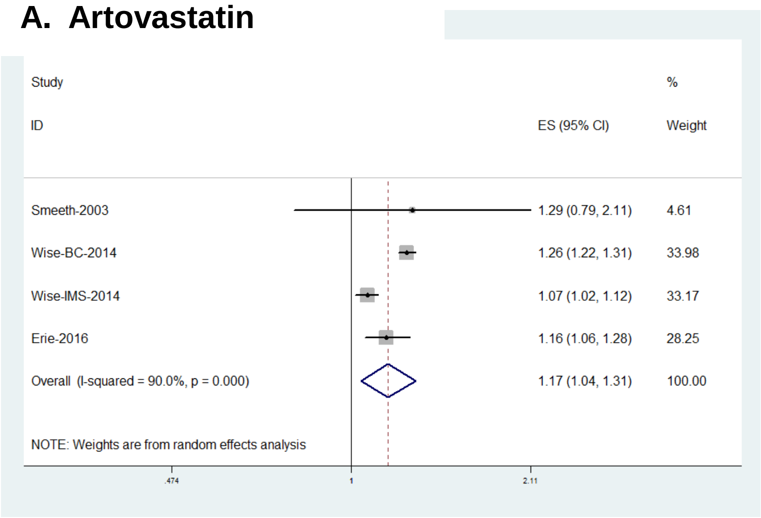

B. Fluvastatin

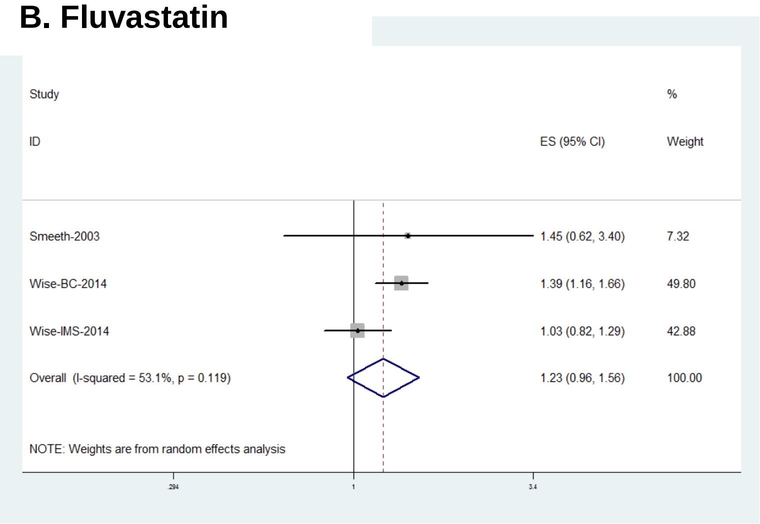

C. Lovastatin

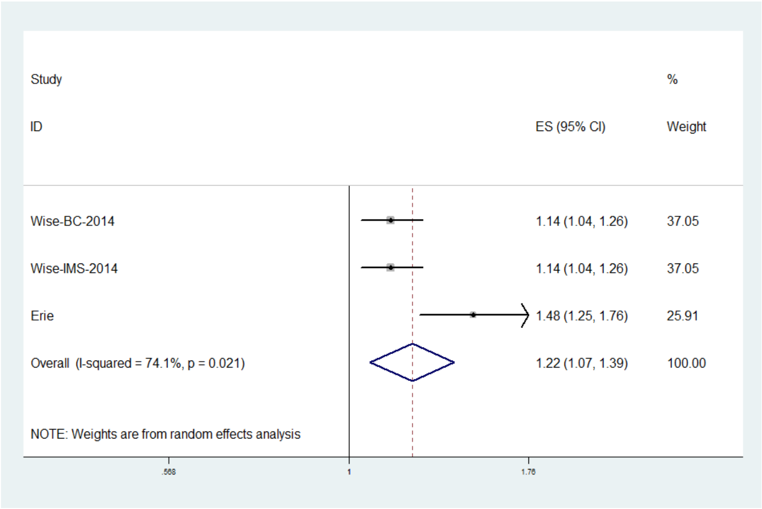

D. Pravastatin

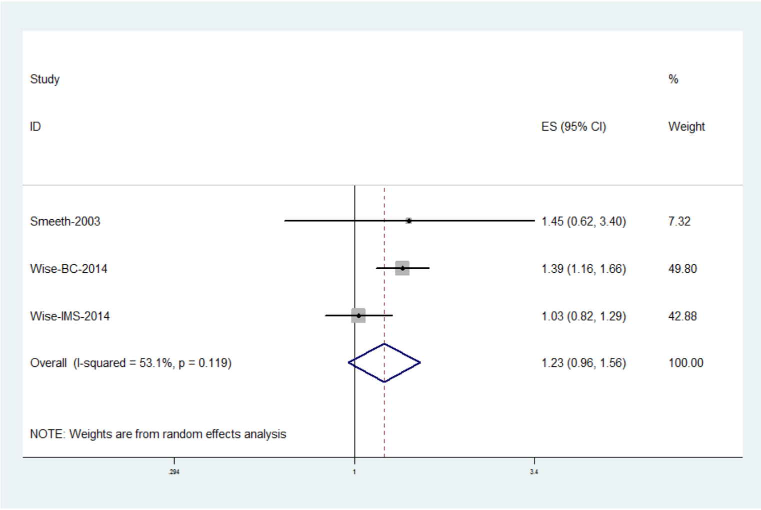

E. Rosuvastatin

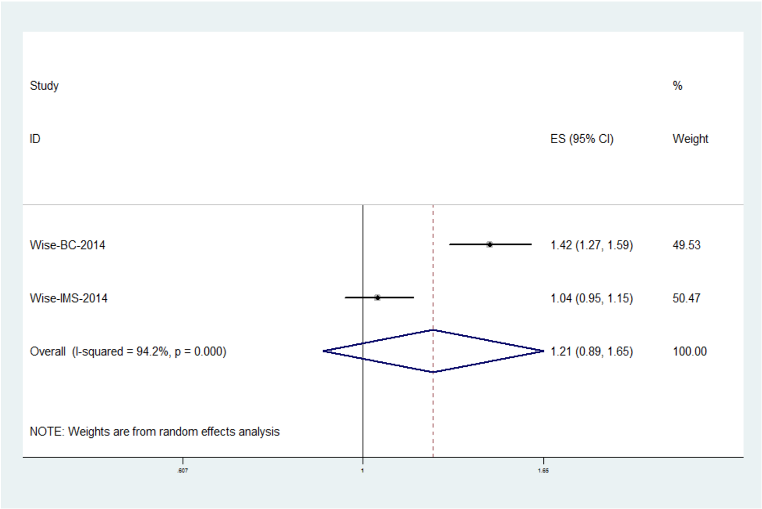

F. Simvastatin

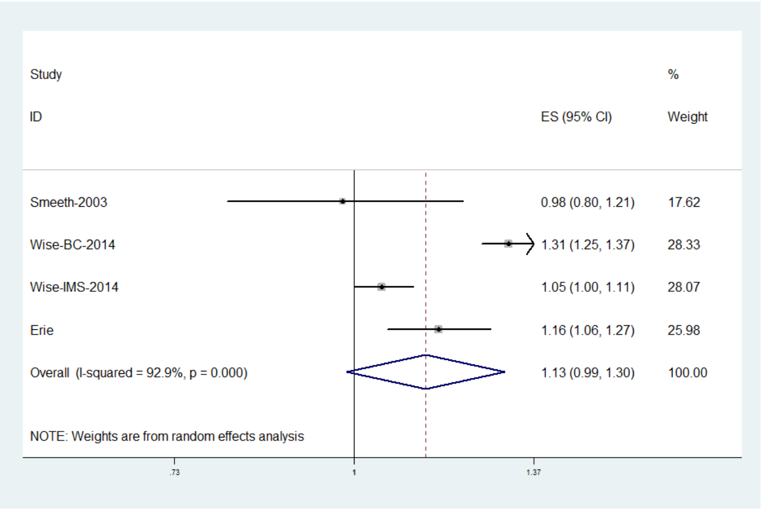

Figure S20. Subgroup analysis of case-control by outcome

A. Cataract surgery

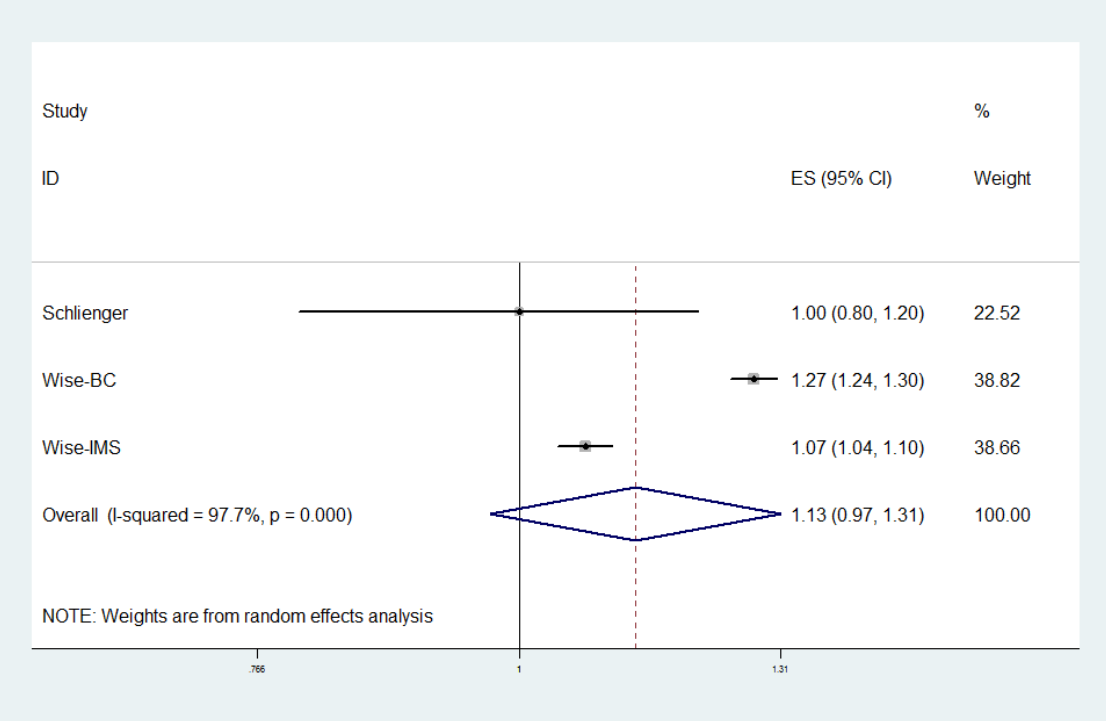

B. Cataract and Surgery

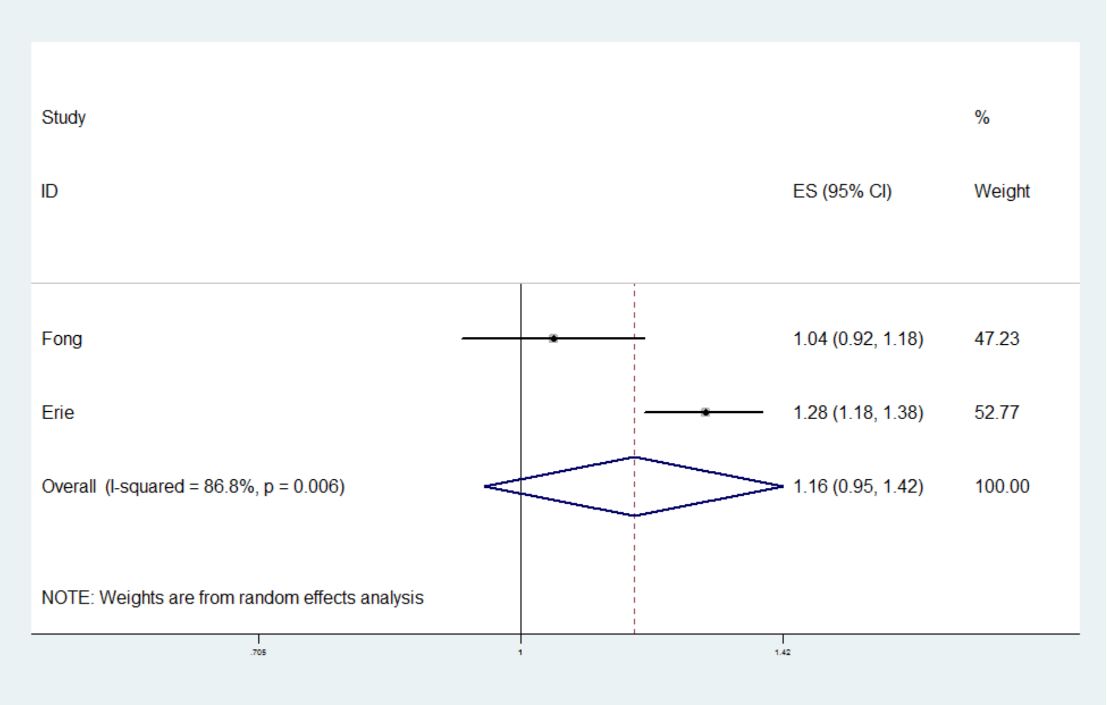

Figure S21.   Subgroup analysis of case-control by CVD

A. CVD included

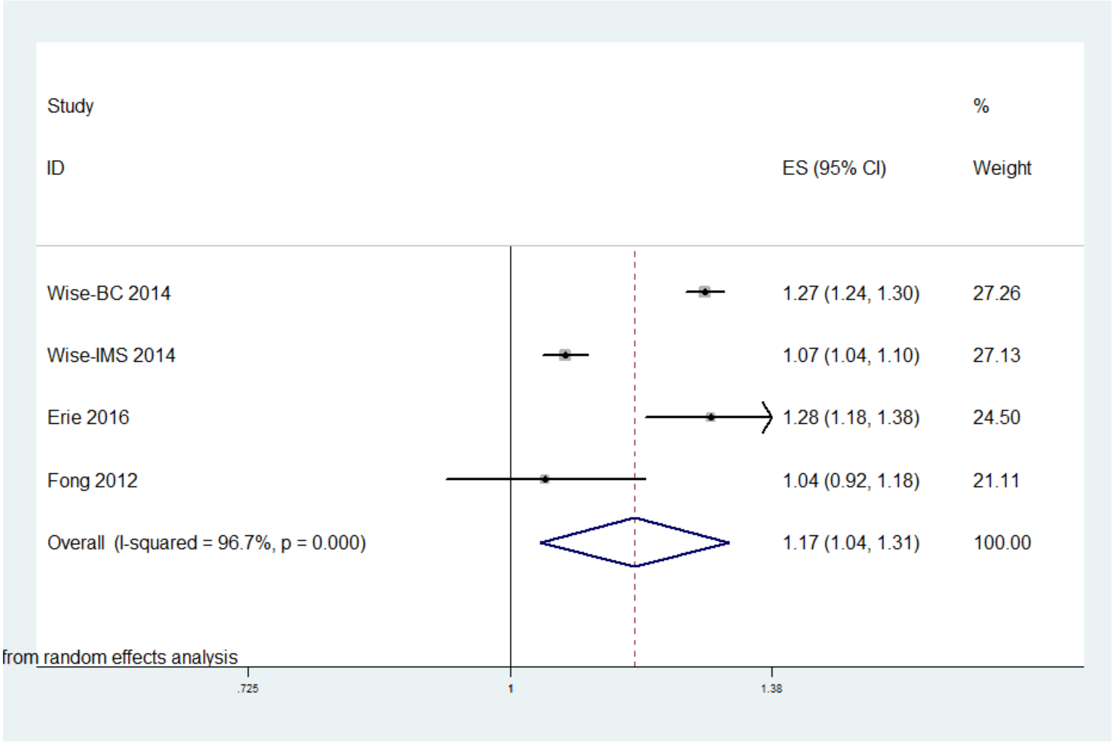

B. CVD missing

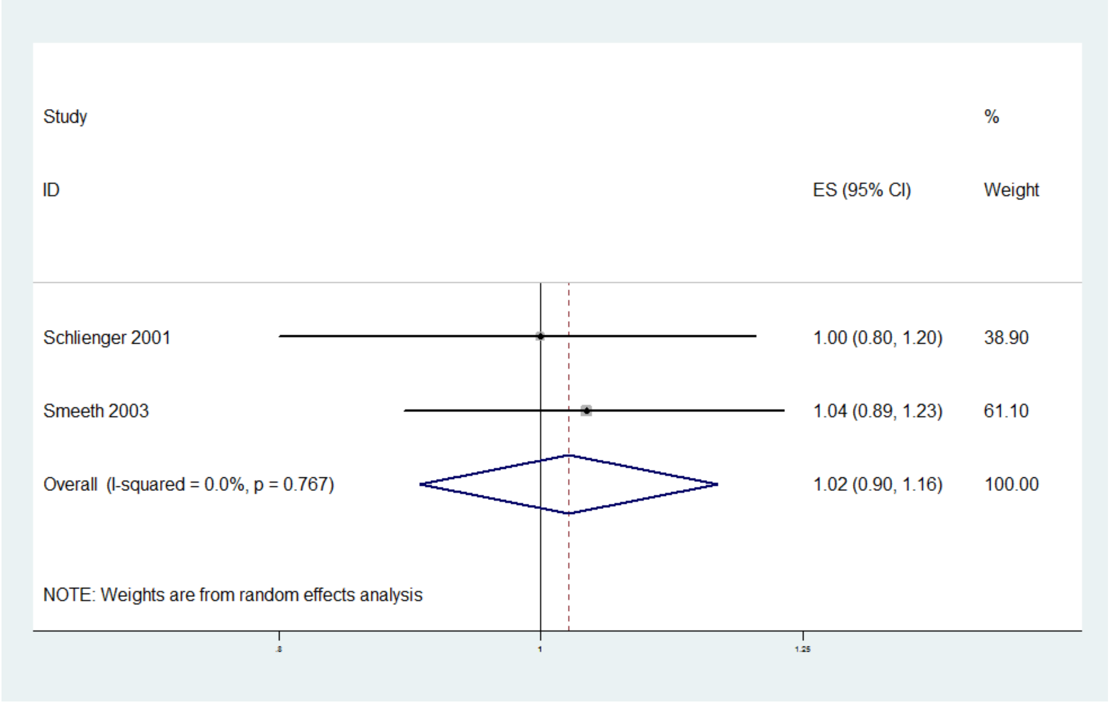

Figure S22. Subgroup analysis of case-control by smoking

A. Smoking included

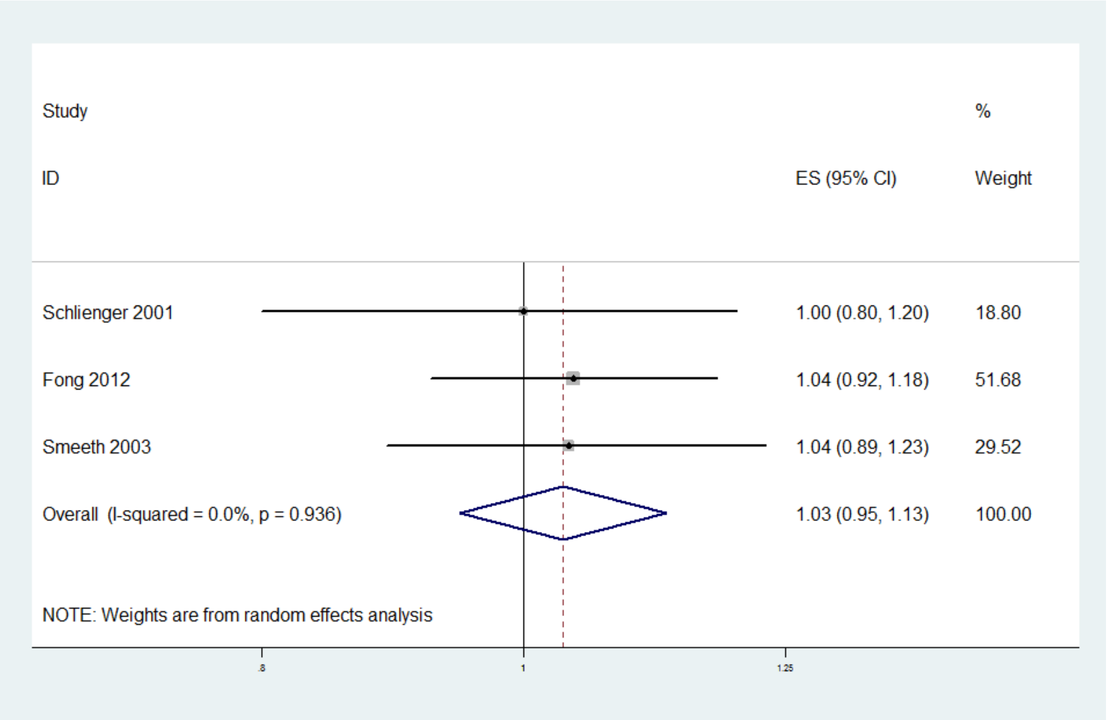

B. Smoking missing

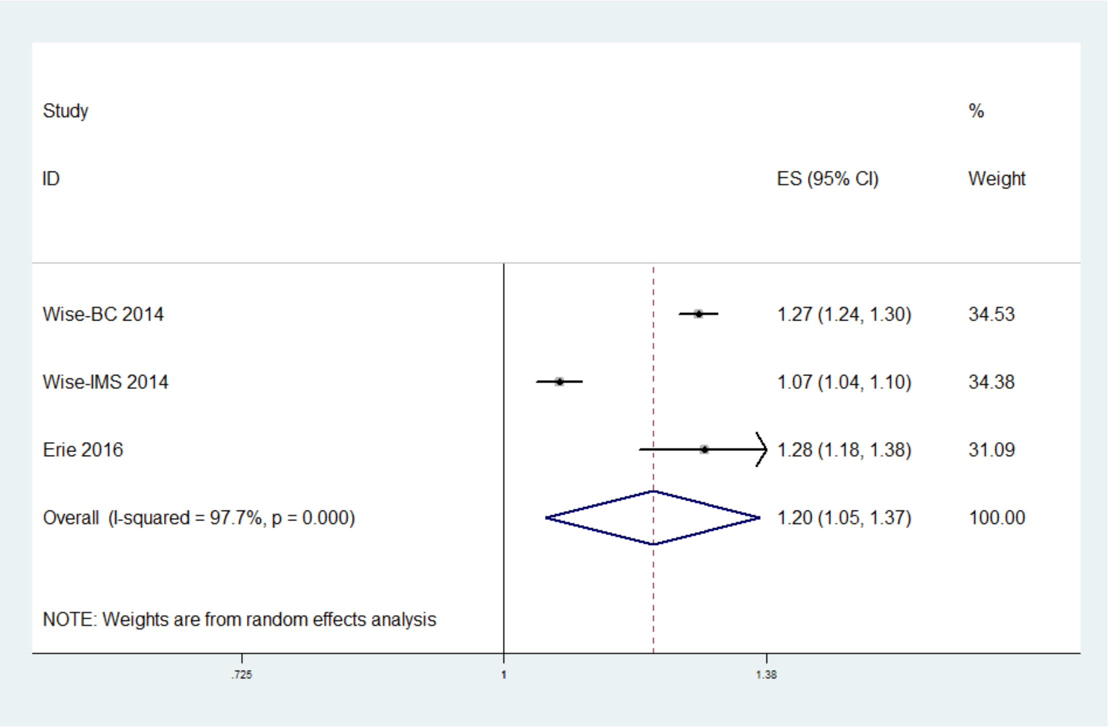

Figure S23. Subgroup analysis by of case-control consultation rate

A. Consultation rate included

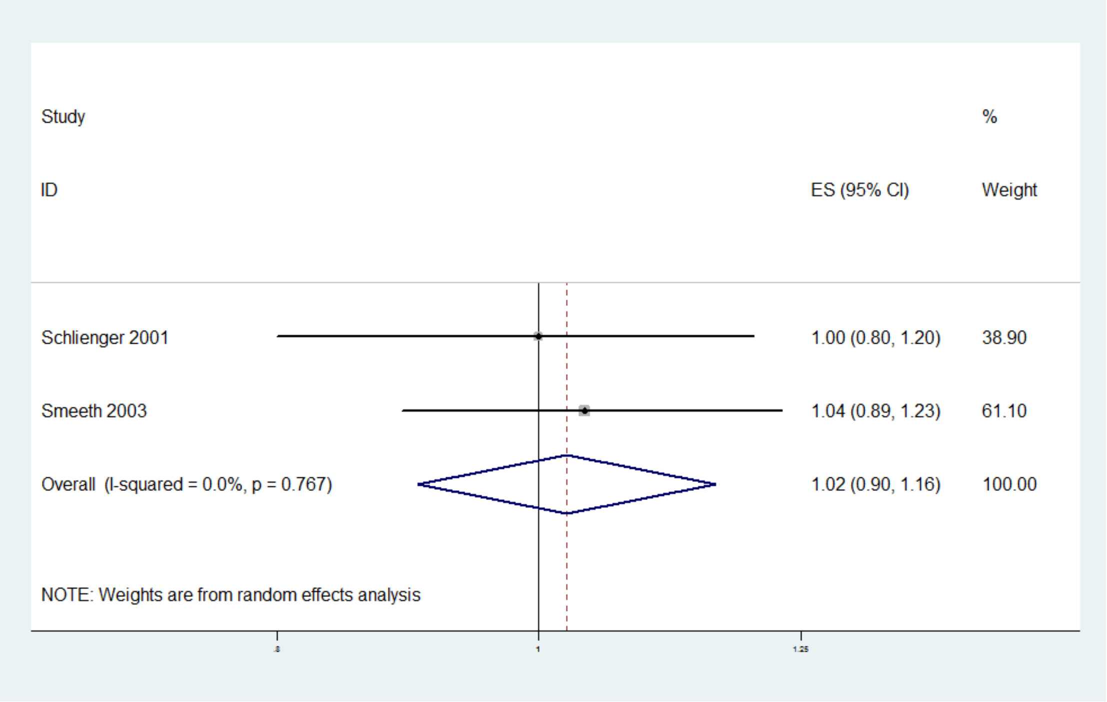

B. Consultation rate missing

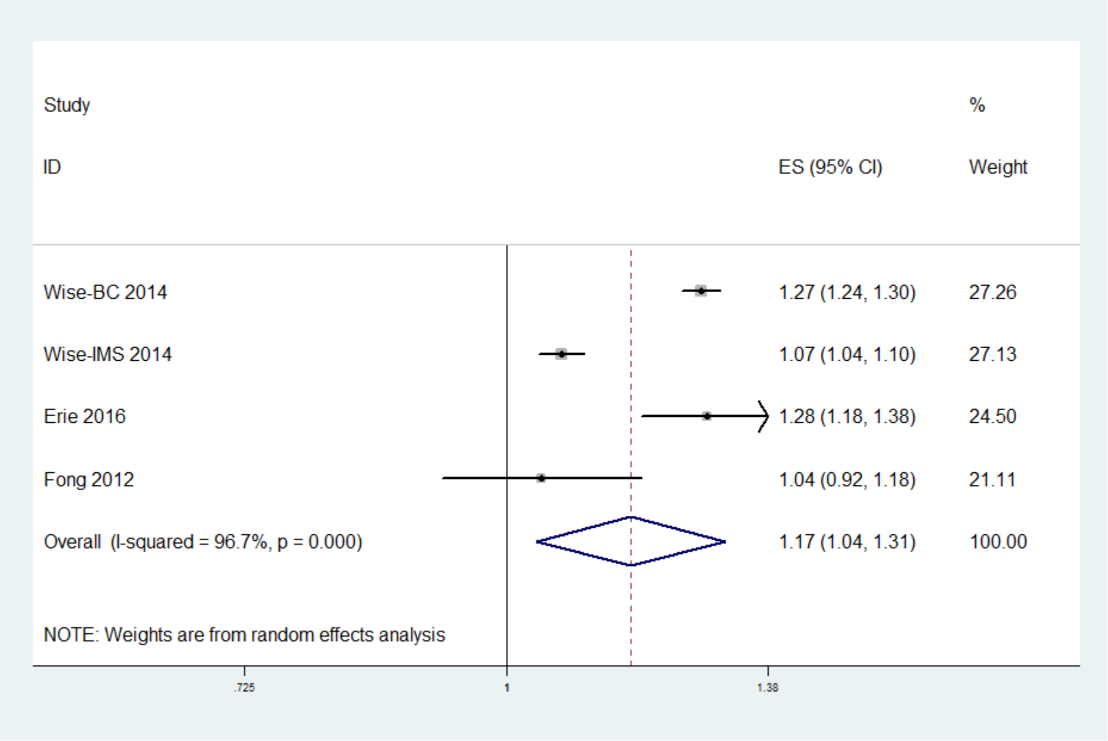

Figure S24. Subgroup analysis of case-control by diabetes (Diabetes included)

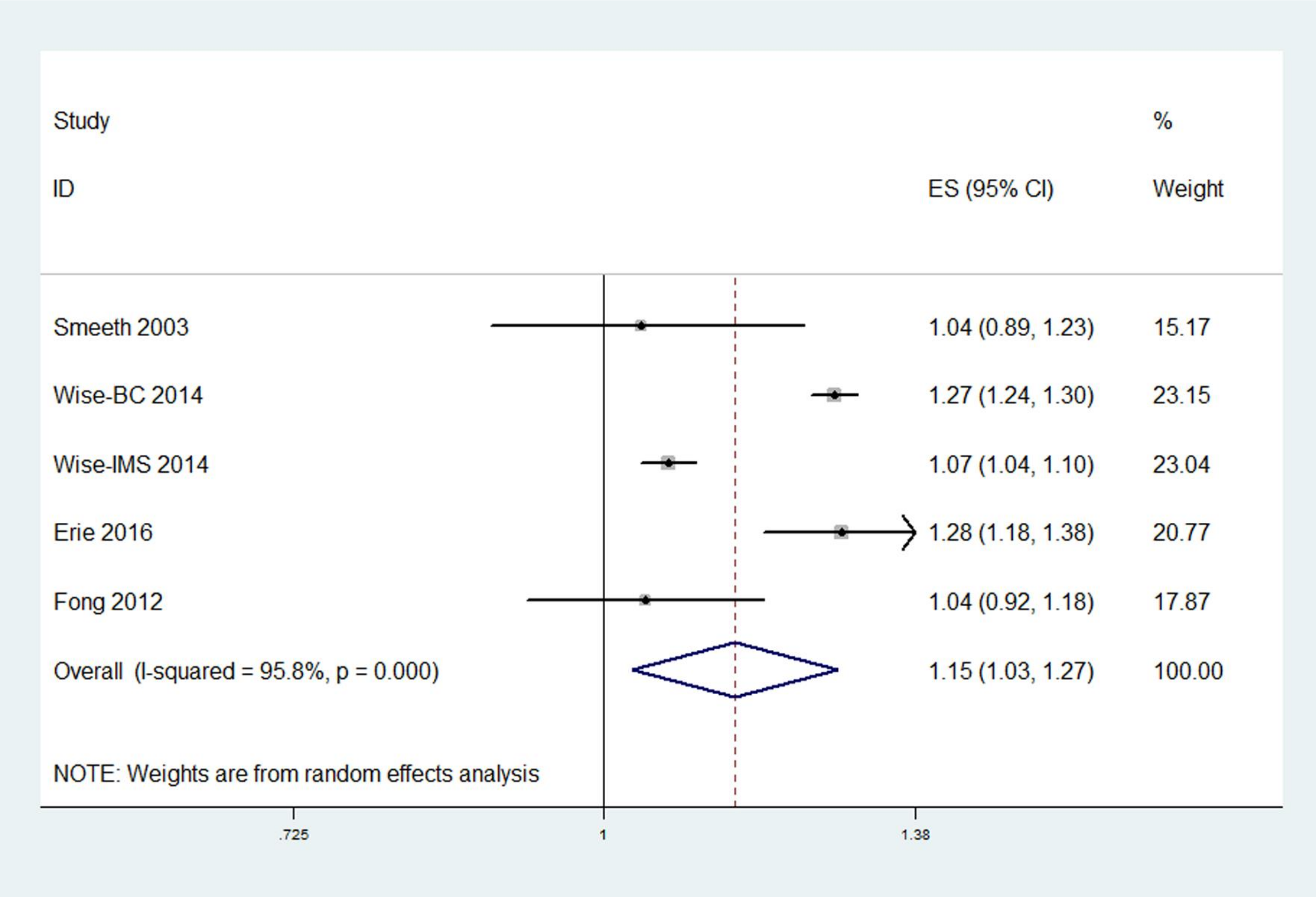

Figure S25. Subgroup analysis of case-control by hypertension

A. Hypertension included

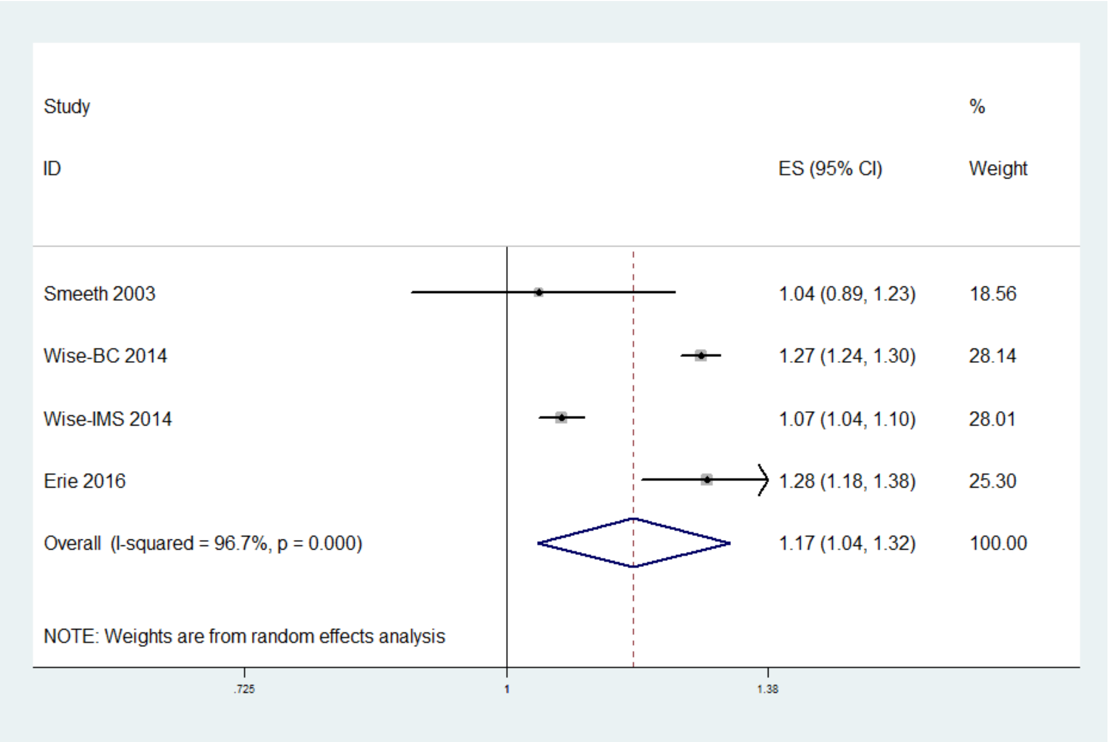

B. Hypertension missing

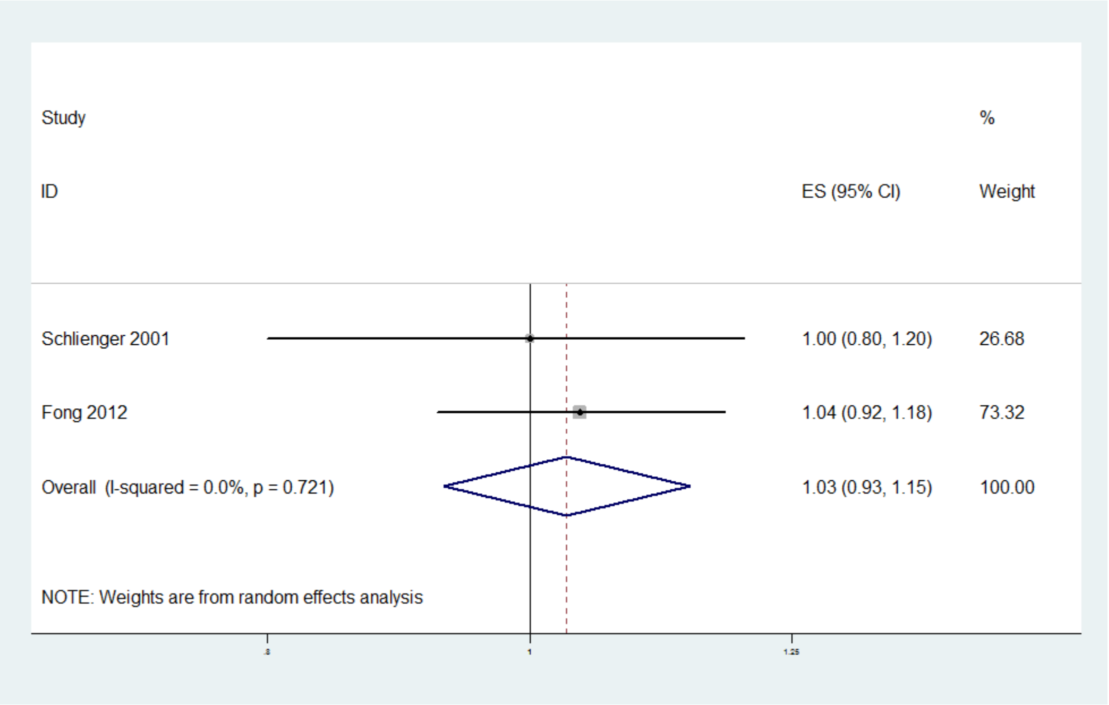

Figure S26. Subgroup analysis of case-control by age (Older than 60)

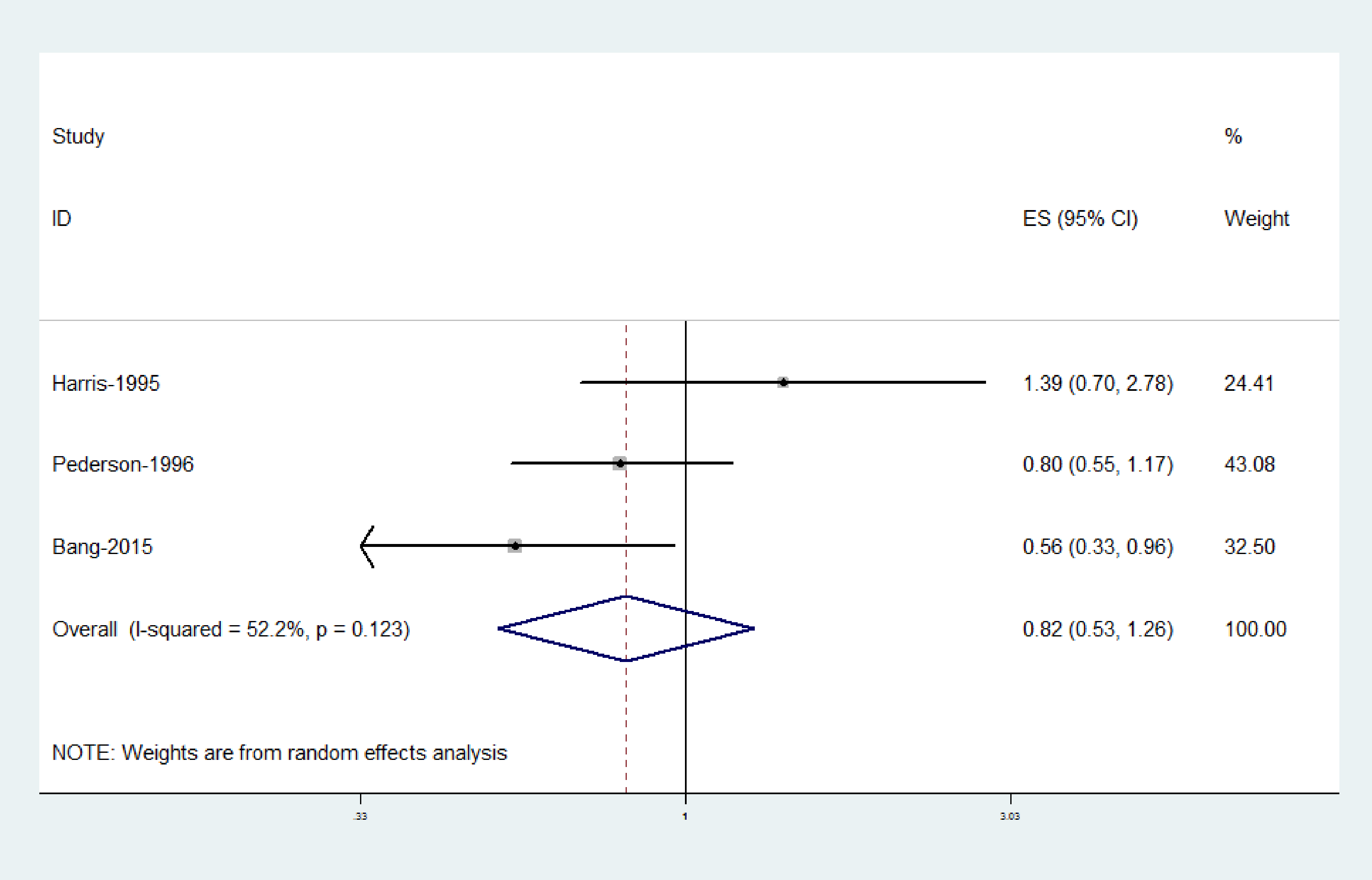

Figure S27. Subgroup analysis of case-control by age (including Spence's)

A. Older than 60

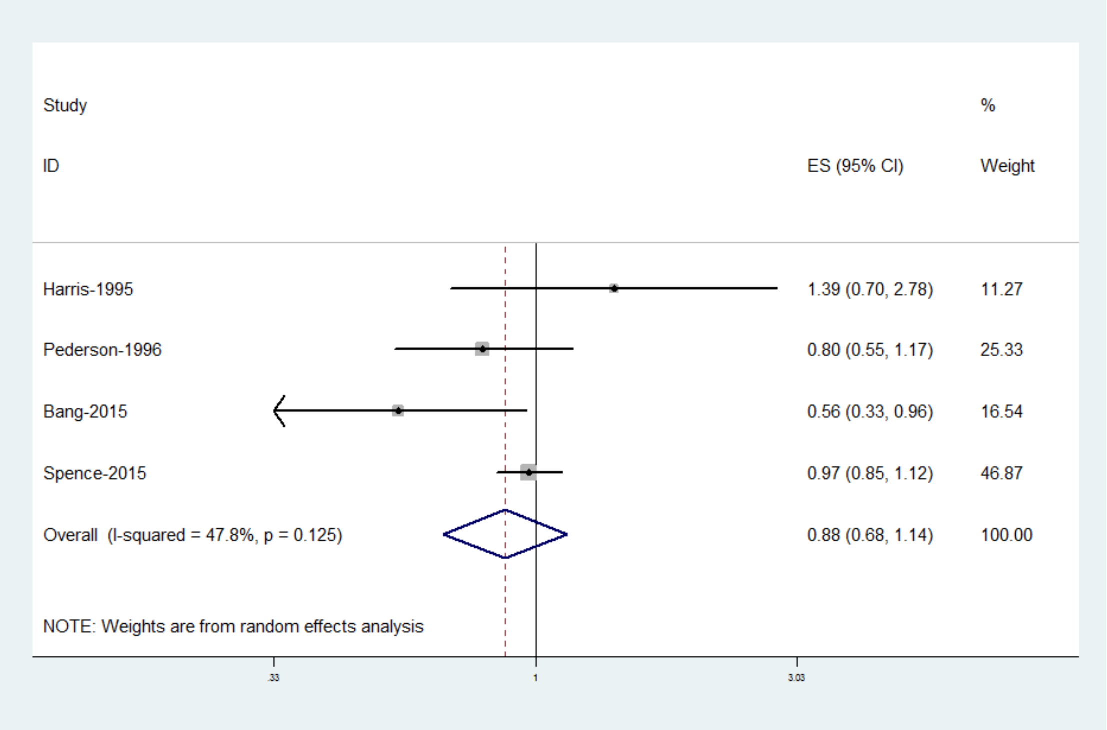

B. Younger than 60

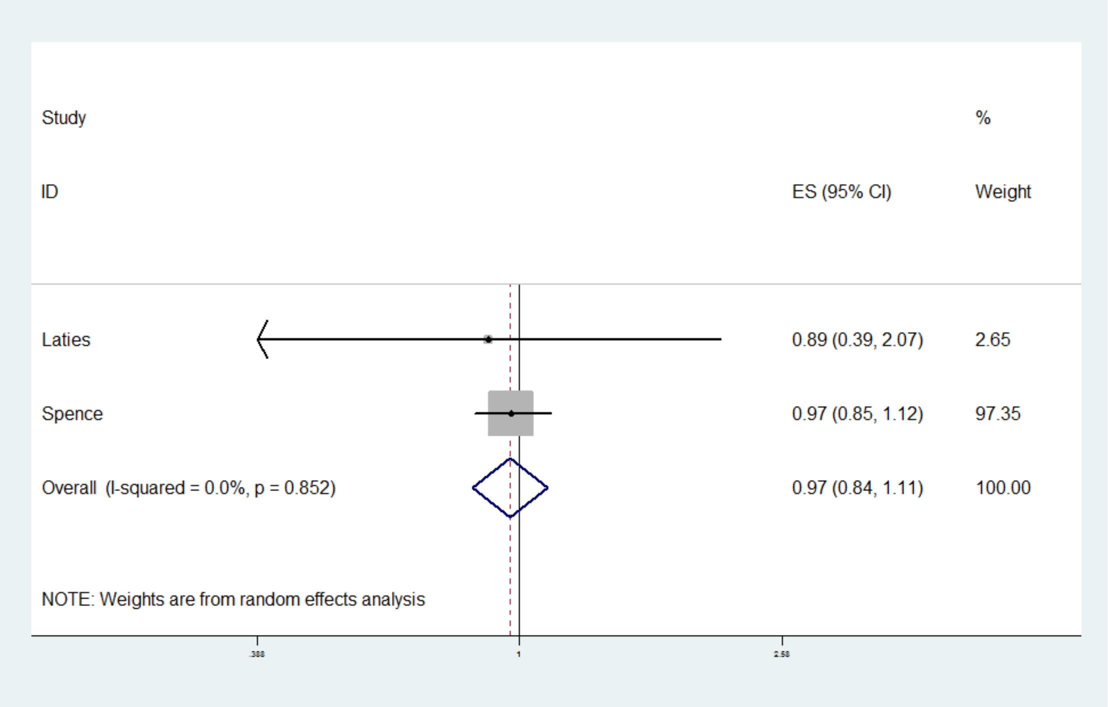

Figure S28. Subgroup analysis of case-control by follow-up duration

A More than 5 years

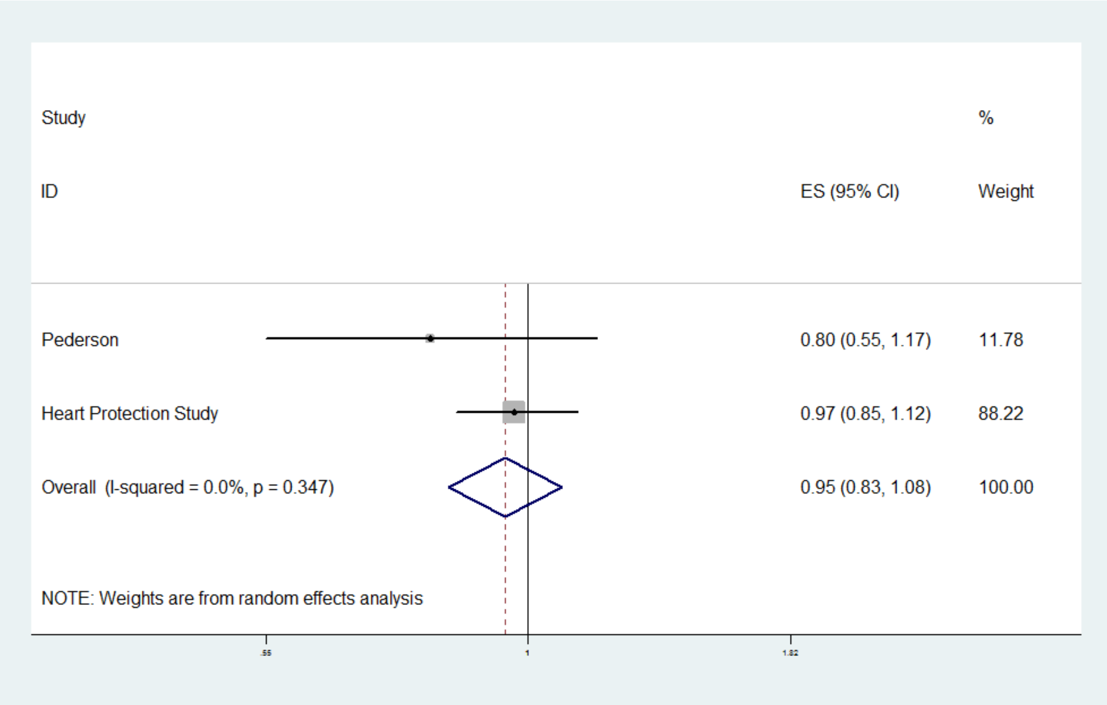

B No more than 5 years

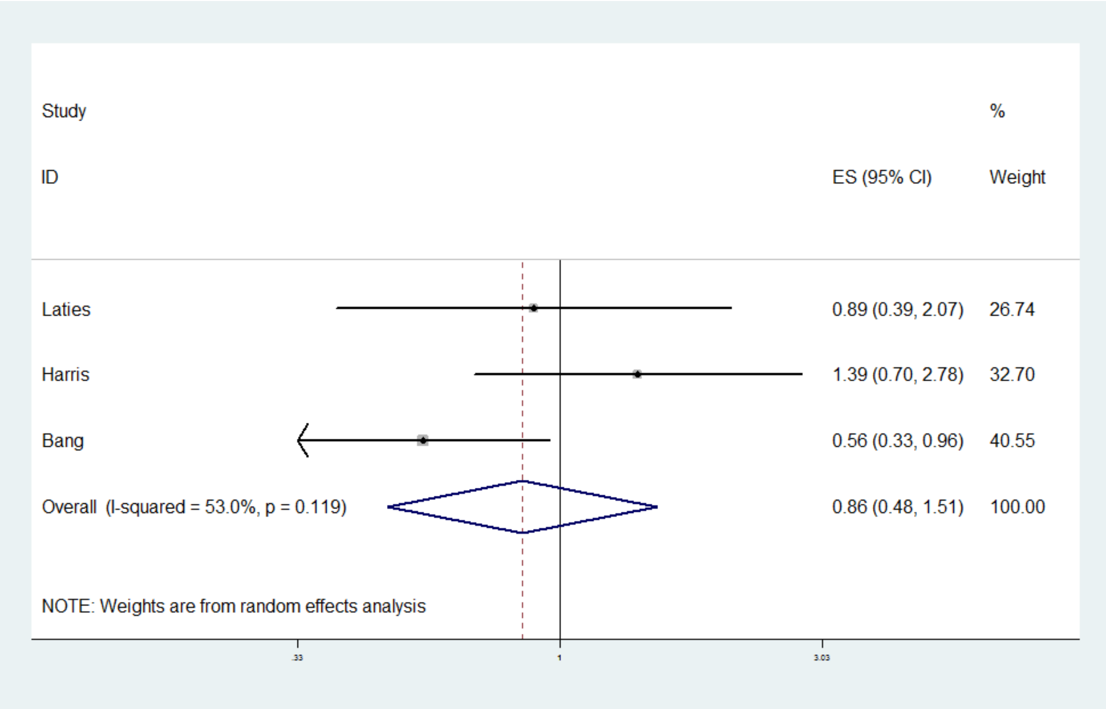

Reference

1. Schlienger RG, Haefeli WE, Jick H, Meier CR: Risk of cataract in patients treated with statins. *Arch Intern Med* 2001, 161:2021-2026.
2. Smeeth L, Hubbard R, Fletcher AE: Cataract and the use of statins: a case-control study. *QJM* 2003, 96:337-343.
3. Fong DS, Poon KY: Recent statin use and cataract surgery. *Am J Ophthalmol* 2012, 153:222-228 e221.
4. Wise SJ, Nathoo NA, Etminan M, Mikelberg FS, Mancini GB: Statin use and risk for cataract: a nested case-control study of 2 populations in Canada and the United States. *Can J Cardiol* 2014, 30:1613-1619.
5. Erie JC, Pueringer MR, Brue SM, Chamberlain AM, Hodge DO: Statin Use and Incident Cataract Surgery: A Case-Control Study. *Ophthalmic Epidemiol* 2016, 23:40-45.
6. Klein BE, Klein R, Lee KE, Grady LM: Statin use and incident nuclear cataract. *Jama* 2006, 295:2752-2758.
7. Tan JS, Mitchell P, Rochtchina E, Wang JJ: Statin use and the long-term risk of incident cataract: the Blue Mountains Eye Study. *Am J Ophthalmol* 2007, 143:687-689.
8. Hippisley-Cox J, Coupland C: Unintended effects of statins in men and women in England and Wales: population based cohort study using the QResearch database. *BMJ* 2010, 340:c2197.
9. Waudby CJ, Berg RL, Linneman JG, Rasmussen LV, Peissig PL, Chen L, McCarty CA: Cataract research using electronic health records. *BMC Ophthalmol* 2011, 11:32.
10. Lai CL, Shau WY, Chang CH, Chen MF, Lai MS: Statin use and cataract surgery: a nationwide retrospective cohort study in elderly ethnic Chinese patients. *Drug Saf* 2013, 36:1017-1024.
11. Leuschen J, Mortensen EM, Frei CR, Mansi EA, Panday V, Mansi I: Association of statin use with cataracts: a propensity score-matched analysis. *JAMA Ophthalmol* 2013, 131:1427-1434.
12. Laties AM, Shear CL, Lippa EA, Gould AL, Taylor HR, Hurley DP, Stephenson WP, Keates EU, Tupy-Visich MA, Chremos AN: Expanded

clinical evaluation of lovastatin (EXCEL) study results. II. Assessment of the human lens after 48 weeks of treatment with lovastatin. *The American journal of cardiology* 1991, 67:447-453.

13. Harris ML, Bron AJ, Brown NA, Keech AC, Wallendszus KR, Armitage JM, MacMahon S, Snibson G, Collins R: Absence of effect of simvastatin on the progression of lens opacities in a randomised placebo controlled study. Oxford Cholesterol Study Group. *The British journal of ophthalmology* 1995, 79:996-1002.

14. Pedersen TR, Berg K, Cook TJ, Faergeman O, Haghfelt T, Kjekshus J, Miettinen T, Musliner TA, Olsson AG, Pyorala K *et al*: Safety and tolerability of cholesterol lowering with simvastatin during 5 years in the Scandinavian Simvastatin Survival Study. *Arch Intern Med* 1996, 156:2085-2092.

15. Bang CN, Greve AM, La Cour M, Boman K, Gohlke-Barwolf C, Ray S, Pedersen T, Rossebo A, Okin PM, Devereux RB *et al*: Effect of Randomized Lipid Lowering With Simvastatin and Ezetimibe on Cataract Development (from the Simvastatin and Ezetimibe in Aortic Stenosis Study). *The American journal of cardiology* 2015, 116:1840-1844.

16. Group HPSC: MRC/BHF Heart Protection Study of cholesterol lowering with simvastatin in 20 536 high-risk individuals: a randomised placebocontrolled trial. *The Lancet* 2002, 360:7-22.
